# Anticooperativity of Multiple Halogen Bonds and Its Effect on Stoichiometry of Cocrystals of Perfluorinated Iodobenzenes

**DOI:** 10.1021/acs.cgd.2c00077

**Published:** 2022-03-24

**Authors:** Nikola Bedeković, Tomislav Piteša, Mihael Eraković, Vladimir Stilinović, Dominik Cinčić

**Affiliations:** † University of Zagreb, Faculty of Science, Department of Chemistry, Horvatovac 102a, 10000 Zagreb, Croatia; ‡ Ruđer Bošković Institute, Bijenička cesta 54, 10000 Zagreb, Croatia

## Abstract

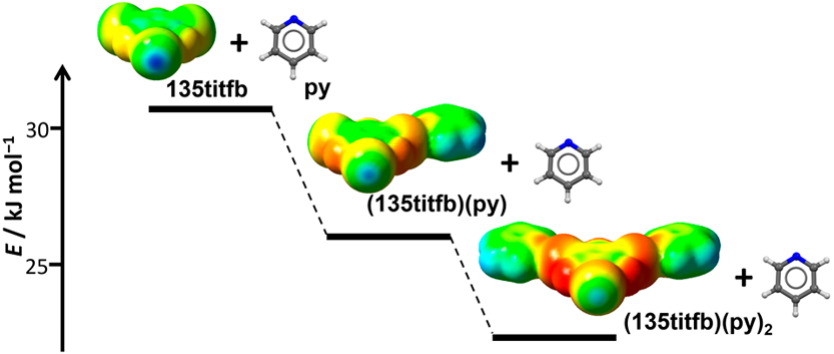

To investigate influences on the topicity of perfluorinated halobenzenes as halogen bond (XB) donors in the solid state, we have conducted a database survey and prepared 18 novel cocrystals of potentially ditopic (**13ditfb**, **14ditfb**) and tritopic (**135titfb**) XB donors with 15 monotopic pyridines. **135titfb** shows high tendency to be mono- or ditopic, but with strong bases it can act as a tritopic XB donor. DFT calculations have shown that binding of a single acceptor molecule on one of the iodine atoms of the XB donor reduces the ESP_max_ on the remaining iodine atoms and dramatically decreases their potential for forming further halogen bonds, which explains both the high occurrence of crystal structures where the donors do not achieve their maximal topicity and the observed differences in halogen bond lengths. Despite the fact that this effect increases with the basicity of the acceptor, when the increase of halogen bond energy due to the basicity of the acceptor compensates its decrease due to the reduction of the acidity of the donor, it enables strong bases to form cocrystals in which a potentially polytopic XB donor achieves its maximal topicity.

## Abstract 2

Consecutive binding of halogen bond acceptors to donor molecules reduces electrostatic potential on free donor atoms, which can affect binding of further acceptor molecules and consequently the stoichiometry of the final product

## Introduction

One of the most fascinating aspects of the study of intermolecular interactions is the effect one interaction can have on other interactions present in the same structure.^1−4^ This effect can be manifested through strengthening (cooperativity) or weakening (anticooperativity) of the interactions involved. This has particularly been studied in the case of hydrogen bonds^5−7^ where (anti)cooperativity and multiple hydrogen bonds have been found to have a profound effect on the properties of liquids,^8−12^ hydration of ions,^13−16^ the structures of biological macromolecules,^17−20^ etc. Cooperativity of hydrogen bonds is most commonly present in hydrogen bonded chains where the atom which is the donor of one hydrogen bond is an acceptor of another (sequence D–H···D–H···), while anticooperativity is most pronounced in systems with multiple hydrogen bonds involving the same donor or the same acceptor atom (sequences D–H···A···H–D and A···H–D–H···A). Anticooperativity of hydrogen bonds can also be observed when two or more different donor (or acceptor) sites are present in the same molecule (D–H···A–R–A···H–D and A···H–D–R–D–H···A), with the effect being reduced with the increase of the separator (R) between the two sites in the molecule.^21^

An interaction similar in many ways to a hydrogen bond is a halogen bond;^22−25^ both are strong and directional interactions with similar ranges of bond energies, and both can vary from purely electrostatic to largely covalent.^26,27^ Besides, in both hydrogen and halogen bonded systems, cooperativity of several bonds can lead to additional stabilization of the halogen (or hydrogen) bonded structure. In halogen bonded systems, this is commonly achieved in type II interhalogen contacts where the same halogen acts as a donor of one halogen bond and acceptor of an orthogonal one,^22,28^ although it has recently been shown that similar stabilization is mostly absent in the case of the triangular halogen bonded synthon.^29^

Anticooperativity in halogen bonded systems has also been demonstrated in the case of bifurcated halogen bonds with multiple acceptors interacting with the same donor (A···X···A) and less so when multiple donors interact with the same acceptor (D–X···A···X–D).^30,31^ Also, there have been strong indications that when the donor halogen atom is surrounded by additional electron density orthogonal to the halogen bond this does decrease the bond strength.^25^ However, the effect of multiple halogen bonds formed by different halogens on the same molecule (i.e., in structures comprising the A···X–D–R–D–X···A halogen bonded sequence) has remained an unresolved question.

In order to examine this point, a detailed study of (potentially) polytopic halogen bond donors (i.e., donors with multiple halogen atoms, which can potentially bind more than one XB acceptor molecule) was necessary, as here the anticooperativity of multiple halogen bonds formed by different halogens on the same molecule could prevent the formation of the maximum number of possible halogen bonds (i.e., prevent the XB donor molecule to achieve its maximal topicity). The obvious choice of compounds which can be used for such a study is perhalogenated hydrocarbons, the most commonly used organic halogen bond donors (in particular, the *ortho*-, *meta*-, and *para*-diiodotetrafluorobenzene—**12ditfb**, **13ditfb**, and **14ditfb** respectively). Of these, the *ortho* isomer is somewhat inappropriate because of possible steric hindrance upon binding of two Lewis base molecules on neighboring iodine atoms. The most commonly used halogen bond donor from this group is the **14ditfb** originally introduced in 2000 by Metrangolo et al.^32^ which has shown to be a very reliable ditopic halogen bond donor, commonly forming two halogen bonds.^33−38^ As opposed to **12ditfb** and **14ditfb**, the third member of this group **13ditfb** was introduced much later (2017).^39^ Although it also is a potentially ditopic halogen bond donor, it has often been found to form only a single halogen bond in crystal structures.^33^

Another especially interesting compound is a potentially tritopic halogen bond donor 1,3,5-triiodo-2,4,6-trifluorobenzene **135titfb**. An early attempt to use **135titfb** as a tritopic halogen bond donor was performed by van der Boom and co-workers^40^ who attempted to produce two-dimensional halogen bonded sheets by cocrystallizing it with ditopic bipyridyl acceptors. They observed that, rather than forming the expected 2:3 cocrystals, the molecules have assembled into halogen bonded chains of 1:1 stoichiometry, where each **135titfb** formed only two halogen bonds. Consecutive binding of the pyridine molecules to the available donor atoms of **135tiftb** was investigated also by computational methods, which have shown significant reduction in bond energies and increase in bond lengths as consecutive acceptor molecules were bonded to the **135titfb** molecule (in the second and third step, the bond energy was reduced by 17% and 14% respectively, and the bond length increased by about 1% in each step). The observed reduction of the halogen bond donor potential of iodine atoms has led to the conclusion that **135titfb** was unlikely to act as a tritopic halogen bond donor. However, in 2010, Roper et al. successfully obtained a 3:1 cocrystal of 4-*N*,*N*′-(dimethylamino)pyridine (**dmap**) with the **135titfb**.^41^ In the same study, they also calculated changes in atomic charges on iodine atoms and halogen bond lengths during consecutive binding of ammonia (probe acceptor molecule) to the three donor atoms of **135titfb**. The obtained results have shown that the halogen bond length increases with the number of bonded acceptors, but binding of the probe molecule caused only a negligible decrease in the partial charge on iodine atoms. Their results, contrary to those of van der Boom, seemed to indicate that **135titfb** should quite easily act as a tritopic donor.

**Scheme 1:**
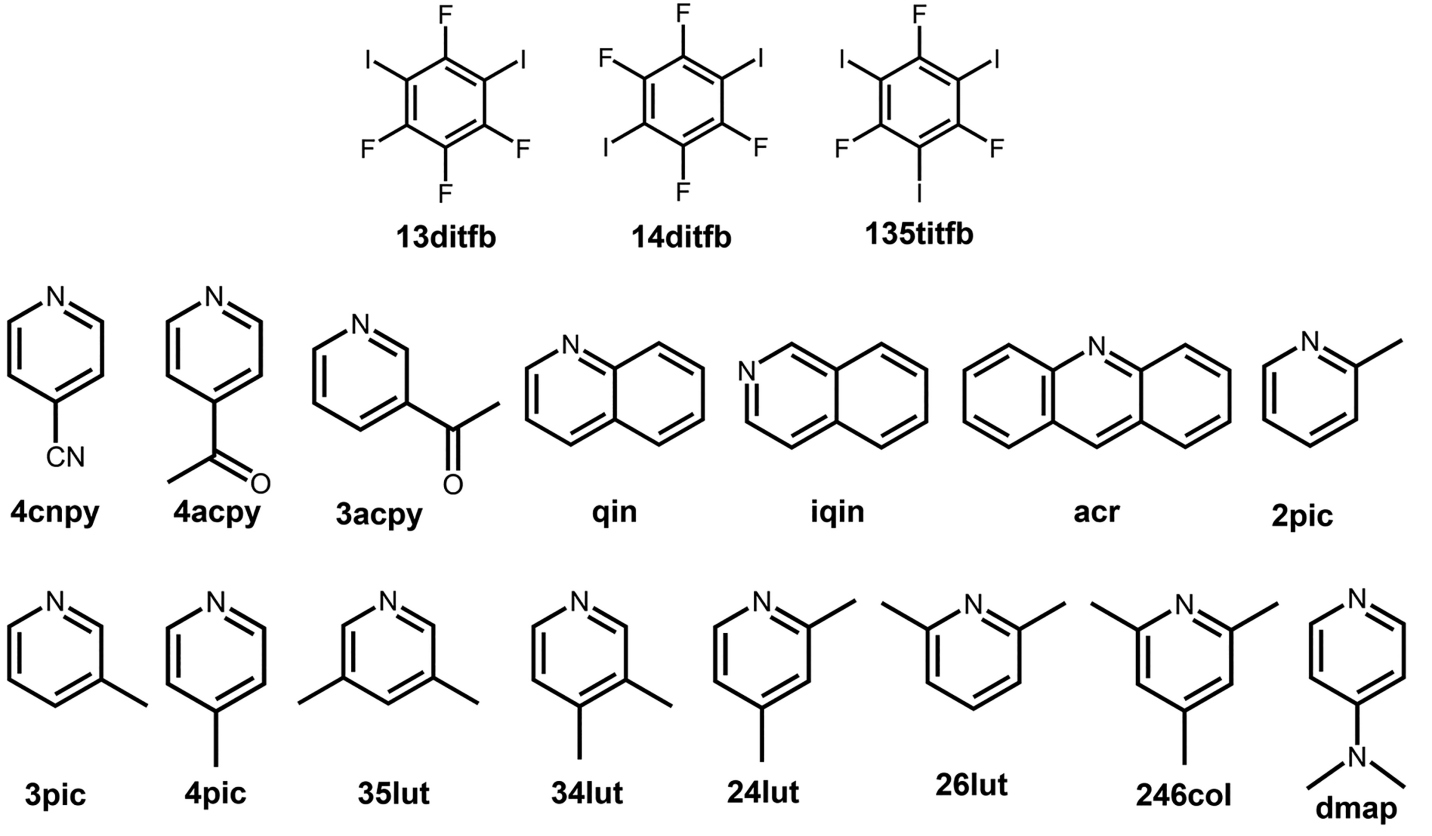
Molecular Diagrams of the Halogen Bond Donor and the Pyridine Derivatives Used in the Study

For the current study, we have decided to investigate the halogen bond donor and acceptor features which can affect the number of bonded acceptor molecules on a certain donor molecule, and how the binding of one acceptor molecule on the donor affects the binding of the second or the third molecule. To exclude both effects of crystal packing and formation of other noncovalent interactions in cocrystals, we have attempted to tackle this question by using **13ditfb**, **14ditfb**, and **135titfb** as halogen bond donors with emphasis on simple, monotopic nitrogen heterocycles (pyridine derivatives, PDs) as halogen bond acceptors in a wide range of basicities (0.87 < p*K*_a_ < 9.60; Scheme 1). In addition, quantum-chemical calculations were employed to rationalize the observed trends. This approach has enabled us to determine how both the topicity and basicity of the acceptor molecules influence the stoichiometry of halogen-bonded cocrystals.

## Results and Discussion

As shown in Table 1, out of the total number of crystal structures deposited in the CSD^42^ including **13ditfb**, **14ditfb**, and **135titfb** as halogen bond donors (Table 1), a large proportion (98%, 81%, and 64%, respectively) are structures of organic compounds containing nitrogen atoms. In the majority of structures which contain the halogen bond donors above and the nitrogen atoms capable of acting as halogen bond acceptors, each donor molecule forms at least one I···N halogen bond (64% of structures with **13ditfb**, 62% of structures with **14ditfb**, and 56% of structures with **135titfb**). However, in compounds in which I···N contact is present, there is a tendency among donors to create multiple halogen bonds. Formation of two halogen bonds is most prominent in structures with **14ditfb** (56% of organic structures with **14ditfb** and nitrogen bases), followed by **13ditfb** (40%) and **135titfb** (30%). Donor **135titfb** can also form three halogen bonds, and this is found in 11% of structures. It follows therefore that out of these three XB donors, only **14ditfb** tends to form the maximal number of I···N contacts in the majority of crystal structures in which such contacts are possible.

**Table 1:** Results of the CSD Survey of Crystal Structures Including **13ditfb**, **14ditfb**, and **135titfb** as Halogen Bond Donors^a^

	**13ditfb**	**14ditfb**	**135titfb**
total number of structures	51	490	153
organic structures with N	50	398	98
one I···N contact	12 (24%)	22 (6%)	15 (15%)
two I···N contacts	20 (40%)	223 (56%)	29 (30%)
three I···N contacts	**–**	**–**	11 (11%)
structures with N_py_	28	166	39
structures with monotopic PD	11	60	20
one I···N_py_ contact	8 (73%)	14 (23%)	14 (70%)
two I···N_py_ contacts	3 (27%)	46 (77%)	5 (25%)
three I···N_py_ contacts	**–**	**–**	1 (5%)
structures with polytopic PD	17	106	19
one I···N_py_ contact	4 (24%)	7 (7%)	5 (26%)
two I···N_py_ contacts	13 (76%)	99 (93%)	13 (69%)
three I···N_py_ contacts	**–**	**–**	1 (5%)

a PD – Pyridine Derivative.

We have further performed a more specific CSD survey restricting nitrogen bases to pyridine derivatives. The results obtained with pyridine acceptors show somewhat different trends to those observed in the survey including all *N*-heterocycles. Among these cocrystals, both **13ditfb** and **14ditfb** mainly act as ditopic donors (in 57% and 87% of structures, respectively), and **135titfb** is almost equally distributed as monotopic and ditopic (49% and 46%, respectively), while in only 5% of cases (i.e., two structures) it forms three halogen bonds. The reason for this discrepancy in the statistics lies in the relatively large number of structures comprising polytopic pyridine derivatives. This can be demonstrated by a further analysis of the data with respect to the number of the pyridine rings in a single molecule of the acceptor. Analysis has shown that there are many more structures with polypyridine acceptors in which **13ditfb**, **14ditfb**, and **135titfb** are ditopic (76%, 93%, and 69%, respectively), than those in which they are monotopic. Conversely, in structures where pyridine is a simple monopyridine (molecule with a single pyridine ring), the probability for the halogen bond donor not to have the maximum possible topicity is much higher (73%, 23%, and 95% for **13ditfb**, **14ditfb**, and **135titfb**, respectively). It is therefore evident that the presence of the polytopic acceptor molecules and polytopic donors has a significant effect on the topicity of the donor in the crystal structures of its cocrystals, favoring higher topicities of the donors. This is particularly pronounced in the case of donors of bent geometry, **13ditfb** and **135titfb** (both predominantly ditopic donors with polytopic acceptors, and monotopic donors with monotopic acceptors), and less so in the case of the linear **14ditfb**. Therefore, in order to investigate the tendencies of the halogen bond donor toward different topicities per se (avoiding the effects of the topicity of the acceptor), simple pyridine derivatives should be used as acceptors. The best pyridine derivatives for such a study would be those which either have no other potential acceptor atoms or have such potential acceptors which are considerably less likely to participate in halogen bonding than the pyridine nitrogen (e.g., oxygen, sulfur, and halogen atoms, or certain nitrogen groups, e.g., cyanide, amide, or aliphatic tertiary amine). We have thus selected eight pyridine derivatives without any competing atoms, covering the p*K*_a_ range of ca. 4.8–7.5. In order to further extend the range of basicities of pyridine derivatives used, we have also included four bases with heteroatoms which are less likely to compete with the pyridine nitrogen as halogen bond acceptors: a highly basic 4-(*N*,*N*′-dimethylamino)pyridine (p*K*_a_ of 9.6) and three weak bases 4-cyanopyridine, 3-acetylpyridine, and 4-acetylpyridine, covering the 2.1–3.8 p*K*_a_ range (Scheme 1, Table 2). While the majority of crystal structures of **13ditfb** and **14ditfb** cocrystals with the selected acceptors have already been determined,^41,43,44^ there were only three structures of cocrystals of **135titfb** with simple pyridines published to date. For the purpose of this study, an additional eleven compounds were prepared in order to expand the data set needed for a more detailed analysis.

**Table 2:** p*K*_a_, Values of Used Acceptors and Donor:Acceptor Ratios in Studied Cocrystals^a^

acceptor	p*K*_a_	**13ditfb**	**14ditfb**	**135titfb**
**4cnpy**	2.10	1:1 (NUBTAI)	1:1 (NUBSEL)	1:1
**4acpy**	3.50	–	1:2	1:2
**3acpy**	3.82	–	1:2	1:1
**qin**	4.85	1:2	1:2	1:1
**iqin**	5.41	1:1	1:2	1:2
**acr**	5.58	1:1	1:2 (VOMHIP)	1:1 (SAJDAL)
**2pic**	5.97	–	1:2	1:3
**3pic**	5.68	–	1:2	1:3
**4pic**	6.02	–	1:2	–
**35lut**	6.24	1:1	1:2	1:3
**34lut**	6.28	1:1	1:2	1:3
**24lut**	6.46	1:1	1:2	1:2
**26lut**	6.72	1:1	**–**	**–**
**246col**	7.48	1:1	1:1	1:3
**dmap**	9.60	1:2 (RUYHOJ)	1:2 (RUYHID)	1:3 (RUYJAX)

a NUBTAI, NUBSEL;^43^ RUYHOJ/RUYHID;^41^ VOMHIP;^44^ SAJDAL.^45^

The tendency of ditopic (**13ditfb**, **14ditfb**) and tritopic (**135titfb**) halogen bond donors to make cocrystals of a certain stoichiometry with the 13 chosen acceptors was investigated by grinding reaction mixtures in 1:2 (ditopic donors) or 1:3 (tritopic donor) stoichiometric ratios (Table 2). Based on the obtained XRPD patterns of the grinding products (Figures S25–S27 in Supporting Information), the formation of new phases has been observed in all the performed reactions. However, additional maxima corresponding to the pure acceptor have been observed in **acr**-**13ditfb**, **acr**-**135titfb**, **4cnpy**-**13ditfb**, **4cnpy**-**14ditfb**, and **4cnpy**-**135titfb** reaction mixtures, which indicated the formation of cocrystals of lower stoichiometry than expected, and the presence of an excess of the acceptor in the reaction mixture. In those five cases, grinding experiments were repeated in 1:2 and 1:1 ratios, and they resulted in the formation of the pure 1:1 cocrystals. Cocrystal screening has also been performed by crystallization from solution, in 1:2 (ditopic donors) or 1:3 stoichiometric ratio (tritopic donor). In this way, we have prepared single crystals and determined crystal structures of seven novel compounds of the ditopic donors—(**13ditfb**)(**24lut**), (**13ditfb**)(**26lut**), (**13ditfb**)(**34lut**), (**14ditfb**)(**3acp**), (**14ditfb**)(**4acp**)_2_, (**14ditfb**)(**24lut**)_2_, and (**14ditfb**)(**34lut**)_2_ and eleven cocrystals of **135titfb** (Table 2). It was found that both crystallization from solution and grinding experiments have resulted in the formation of identical crystal phases in all donor–acceptor combinations.

**Figure 1:**
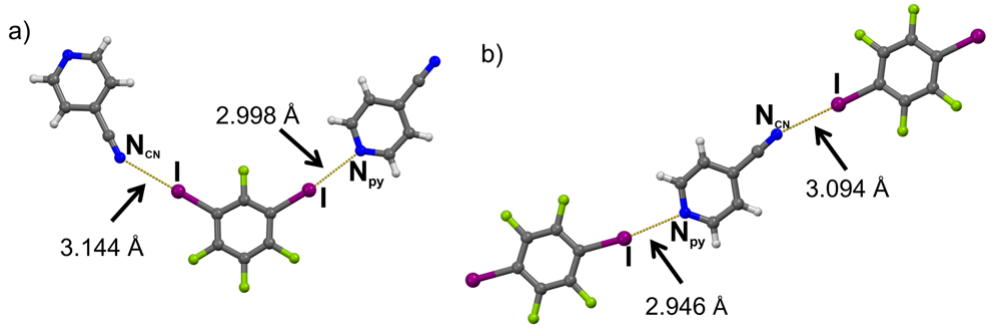
Two types of I···N halogen bonds and their lengths formed in (a) (**13ditfb**)(**4cnpy**) and (b) (**14ditfb**)(**4cnpy**).

Despite the small differences in ESP_max_ values on donor iodine atoms on **13ditfb** (122 kJ mol^–1^) and **14ditfb** (127 kJ mol^–1^), their tendency to form cocrystals as ditopic donors is quite different. The bent **13ditfb** is generally a monotopic donor, with two exceptions—(**13ditfb**)(**qin**)_2_ and (**13ditfb**)(**dmap**)_2_. In both crystal structures the two halogen bonds formed are of different lengths and angles. Out of the three possible potentially ditopic acceptors (**4cnpy**, **3acpy**, **4acpy**) only in the case of **4cnpy** does the second acceptor (cyano nitrogen) participate in an additional XB contact with **13ditfb** (Figure 1a). This contact is considerably longer (*d*(I···NCN) = 3.144(2) Å) than the one between the other iodine and the pyridine nitrogen (*d*(I···N_py_) = 2.998(1) Å;). Unlike **13ditfb**, the linear **14ditfb** is mostly ditopic, with two exceptions: (**14ditfb**)(**4cnpy**) and (**14ditfb**)(**246col**). In the former case, **4cnpy** again acts as ditopic acceptor forming a short I···N_py_ halogen bond (*d*(I···N) = 2.946(8) Å) and a longer halogen bond with cyano nitrogen (*d*(I···N) = 3.094(9) Å; Figure 1b), and resulting in a halogen-bonded chain. In (**14ditfb**)(**246col**), the deviation from the expected topicity is the result of close packing of molecules, which was explained in more detail in our previous study.^33^ In all cocrystals of 1:2 stoichiometry, the **14ditfb** molecule is positioned on the crystallographic inversion center making the two halogen bonds identical.

**Figure 2:**
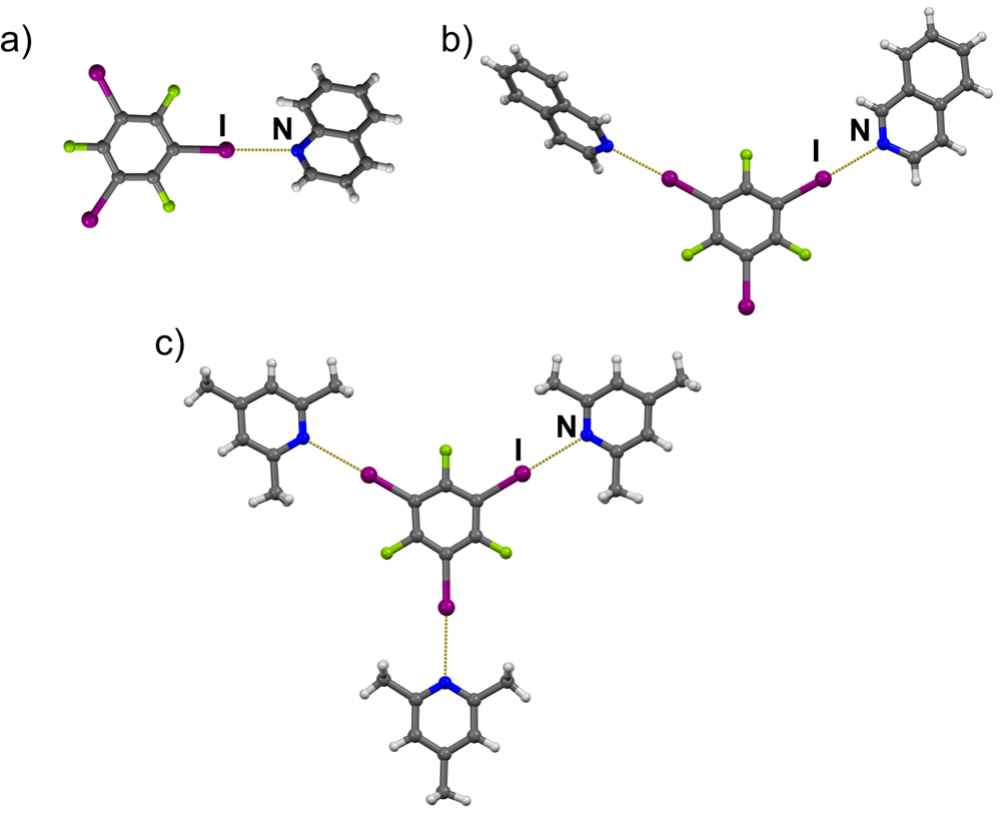
Halogen bonded molecular complexes in (a) (**135titfb**)(**qin**), (b) (**135titfb**)(**iqin**)_2_, and (c) (**135titfb**)(**246col**)_3_.

**Figure 3:**
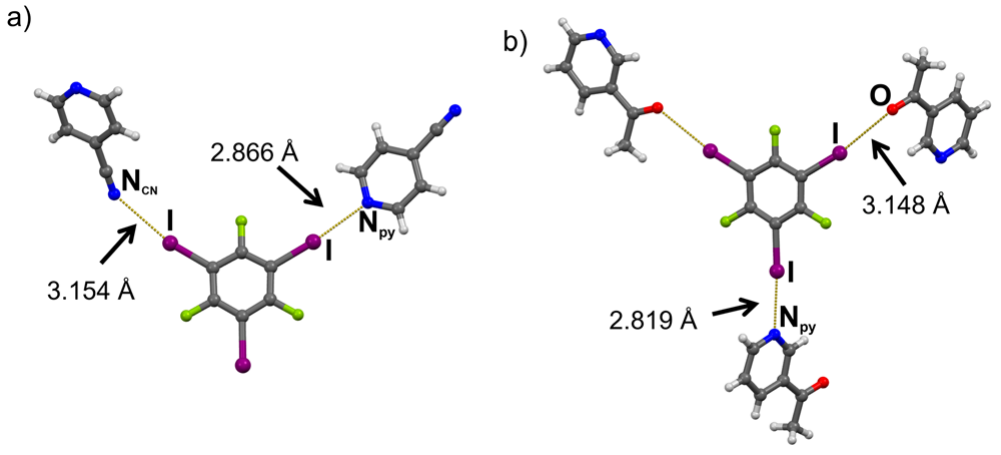
(a) Two types of I···N halogen bonds and their lengths formed in (**135titfb**)(**4cnpy**). (b) I···N and I···O halogen bonds formed in (**135titfb**)(**3acpy**).

A more complex situation has been found among cocrystals of **135titfb**: in four compounds it was found to be monotopic, in three compounds ditopic, and in six of them it formed three I···N_py_ halogen bonds (Table 2, Figure 2). Additional contacts between iodine atoms and either cyano or keto groups (longer than I···N_py_ halogen bonds) have been noticed in crystal structures of (**135titfb**)(**3acpy**) and (**135titfb**)(**4cnpy**) (Figure 3). In (**135titfb**)(**3pic**)_3_ and (**135titfb**)(**246col**)_3_, where **135titfb** has been found to be tritopic, the three I···N_py_ halogen bonds are of different lengths, while in other 1:3 cocrystals, there are also three I···N_py_ contacts of which two are related by symmetry. From the data represented in Table 2, one can notice an interrelation between acceptor basicity and donor topicity observed in the crystal structures of the prepared cocrystals. The vast majority of strong bases (**2pic**, **3pic**, **35lut**, **34lut**, **246col**, and **dmap**) form cocrystals of 1:3 stoichiometry, while weaker bases (**4cnpy**, **3acpy**, **4acpy**, **qin**, **iqin**, **acr**) form cocrystals either of 1:1 or 1:2 stoichiometries. This indicates that there is a significant effect of the basicity of the acceptor on the stoichiometry of the cocrystal formed with **135titfb** (and therefore on the topicity of **135titfb**). It has to be noted that this conclusion does not seem to be valid for **13ditfb** and **14ditfb**, which preferentially form 1:1 and 1:2 cocrystals, respectively, with almost all bases used.

**Figure 4:**
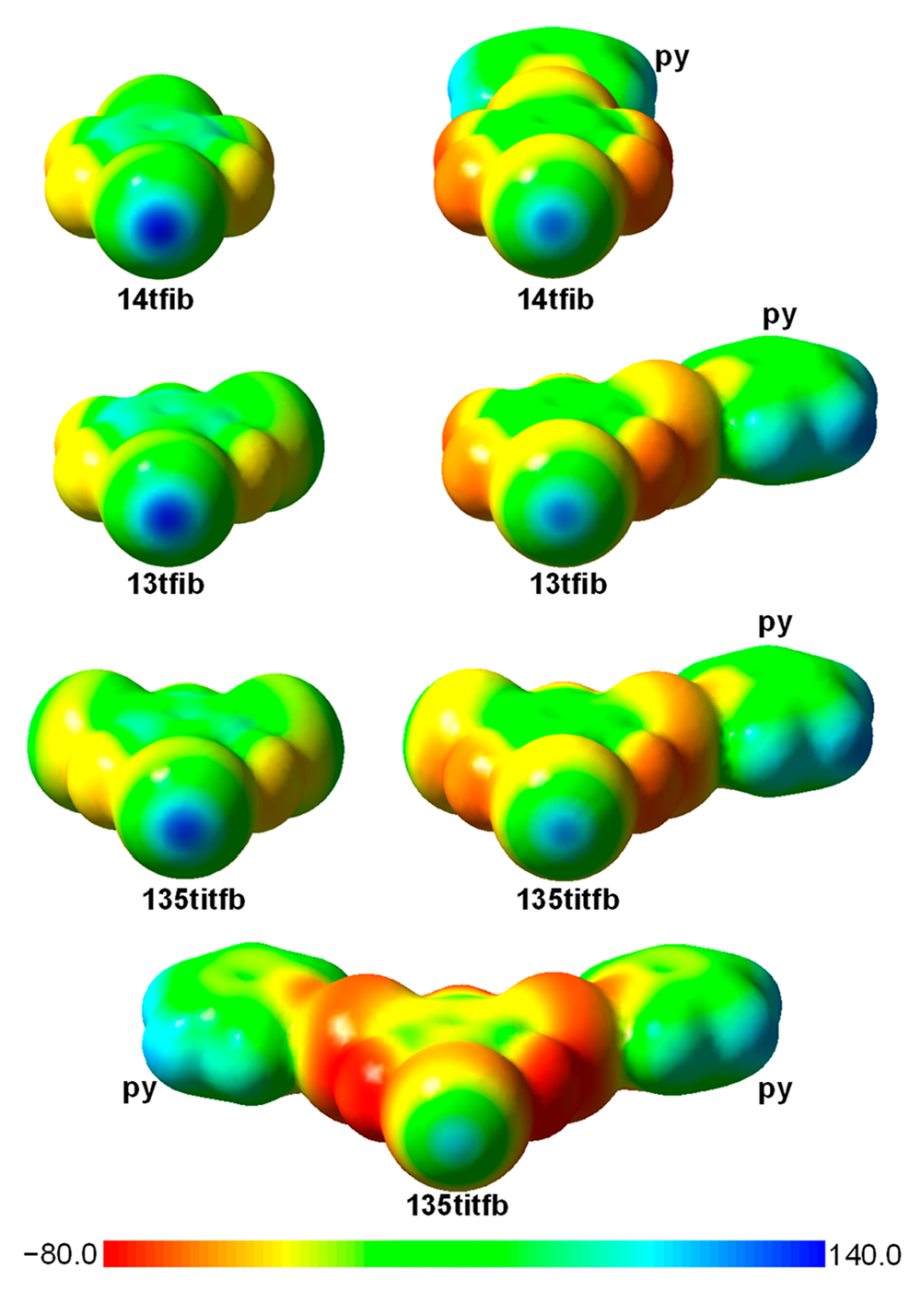
ESP mapped on the electron density isosurface (ρ_el_ = 0.001 au) in systems **14ditfb**, **13ditfb**, **135titfb**, (**14ditfb**)**•**(**py**), (**13ditfb**)**•**(**py**), (**135titfb**)**•**(**py**), and (**135titfb**)**•**(**py**)_2_. Boundaries of ESP values are given in kJ mol^–1^ e^–1^.

**Figure 5:**
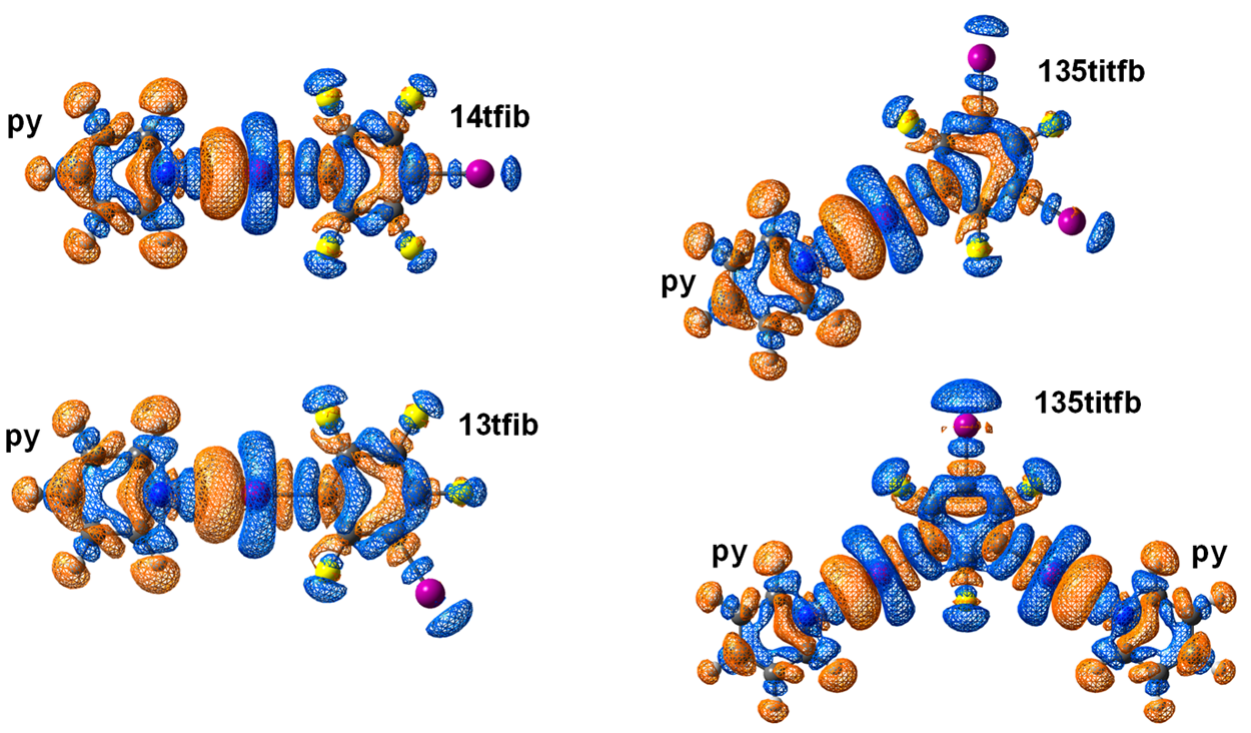
EDD isosurfaces (|Δρ_el_| = 2 × 10^–4^ au) upon binding of a **py** molecule to **14ditfb**, **13ditfb**, and **135titfb**, and upon binding of two **py** molecules to **135titfb**. Blue parts of isosurfaces correspond to the positive and brown parts to the negative value of Δρ_el_.

In order to ascertain whether the differences in the behavior of **13ditfb**, **14ditfb**, and **135titfb** as halogen bond donors are due to their electronic structures, we have performed a series of quantum-chemical computations aimed at observing the differences in the effect of binding of base molecules on each of the studied halogen bond donors. Computations using pyridine as the probe acceptor molecule have shown that binding of a single pyridine molecule on one of the iodine atoms of the XB donor reduces the ESP_max_ (on 0.001 au electron density isosurface; Figure 4) on the remaining iodine atom(s) by 23.0, 23.4, and 21.9 kJ mol^–1^ e^–1^ in **14ditfb**, **13ditfb**, and **135titfb**, respectively (on average by ca. 17%). Binding of a further pyridine molecule on a second iodine atom of **135titfb** reduces the ESP_max_ on the remaining unbonded iodine atom by a further 19.3 kJ mol^–1^ e^–1^. This indicates a dramatic decrease in the potential of the nonhalogen-bonded iodine atom for forming a further halogen bond. This is mirrored by the reduction of halogen bond energies: the binding energies of the first pyridine molecule onto the three donors are 31.4, 30.8, and 30.1 kJ mol^–1^, while for the second pyridine they are 27.8, 26.8, and 26.4 kJ mol^–1^ (for **14ditfb**, **13ditfb**, and **135titfb**, respectively), which corresponds to relative reductions of 11%, 13%, and 12%. Further binding energy of the third pyridine molecule onto **135titfb** (23.4 kJ mol^–1^) is overall reduced by 22%. It should be noted, however, that the overall partial charge of the nonhalogen-bonded iodine atoms changes significantly less: binding of a pyridine molecule on one of the iodine atoms of either donor reduces the NBO charge of the other iodine atom(s) by ca. 0.015 e—a decrease of only 6%. The apparent discrepancy can be explained by referring to the electron density difference (EDD) plots (Figure 5).

These reveal how electron densities in both the donor and the acceptor molecules are ostensibly perturbed by the formation of the halogen bond, primarily about the donor and acceptor atoms (as demonstrated in earlier studies by crystallographic charge density analysis).^26^ By concentrating however on the nonbonding iodine atom, a large increase of electron density can be seen in the σ-hole region of the atom, coupled with a slight decrease of electron density perpendicular to it—particularly visible in the 2:1 complex of **135titfb** (Figure 4). Therefore, while there is a significant decrease of the (positive) ESP on the iodine atom, the change of the total charge is slighter, as the corresponding increase of electron density is partially compensated by a slight increase in the perpendicular direction.

**Scheme 2:**
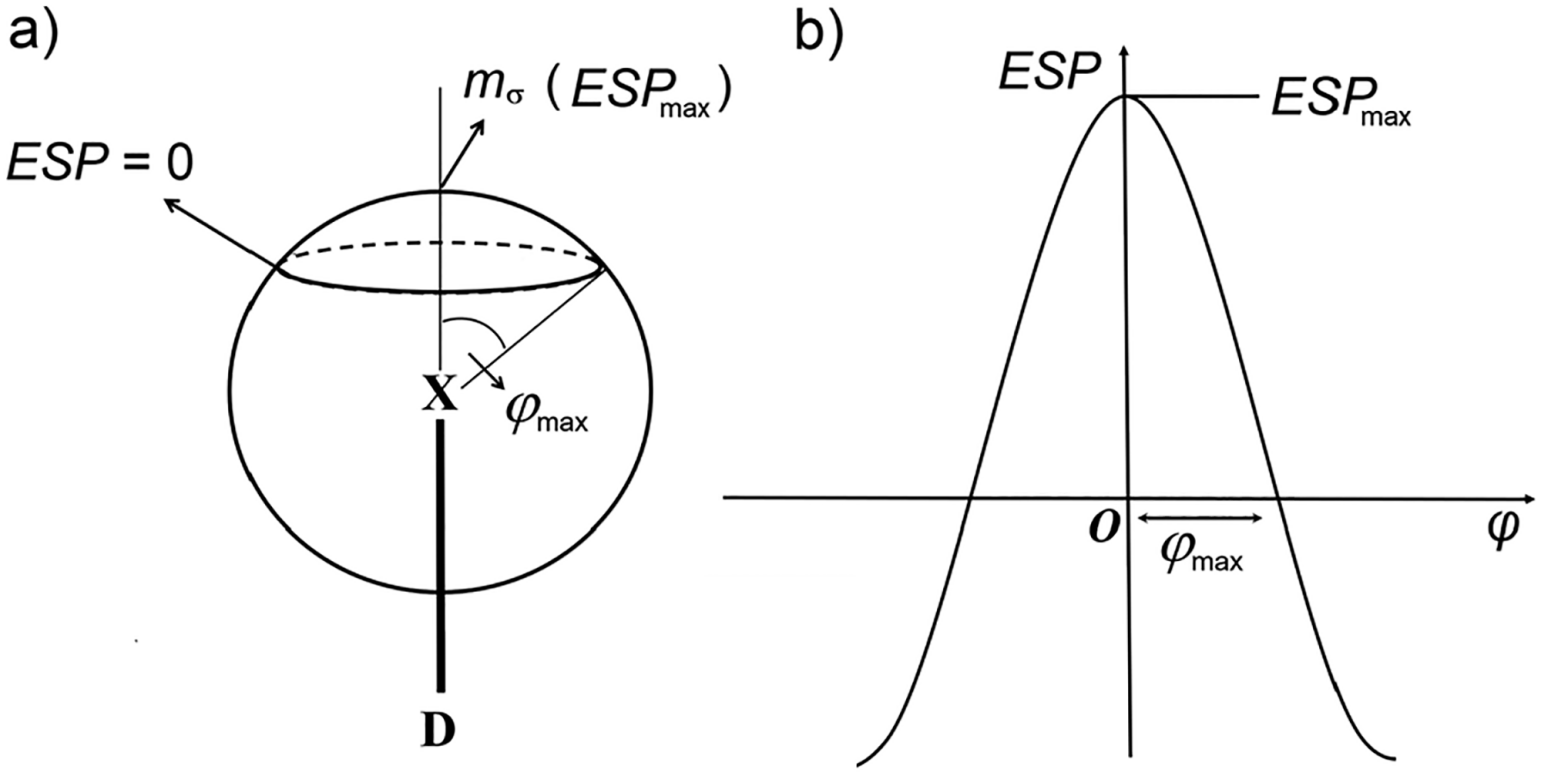
(a) Definition of the Parameters of the σ-Hole on the Halogen Atom (X); (b) Qualitative Representation of ESP on a Halogen Atom in the Plane Containing the D–X Bond As a Function of Angle φ

**Figure 6:**
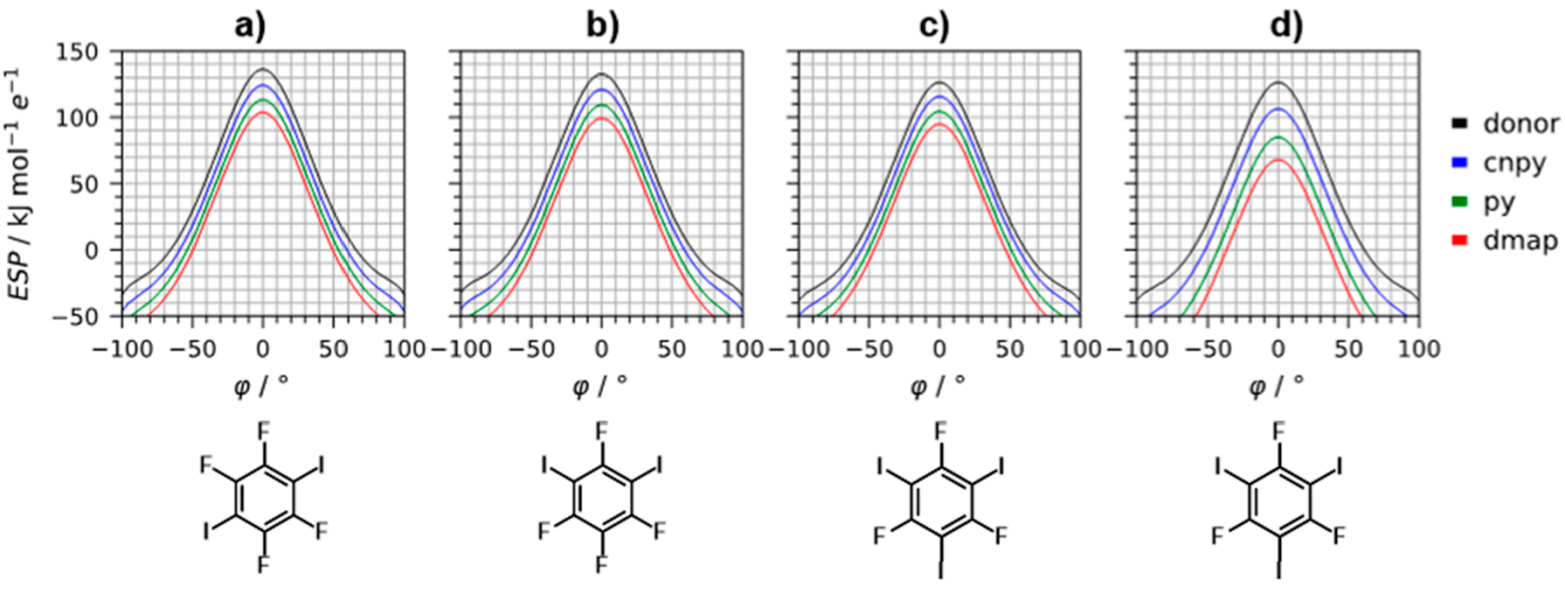
Angular dependence (see Scheme 2 for the definition of the φ angle) of molecular electrostatic potential in the donor-molecule plane on the σ-hole of the free iodine atom, evaluated on the 0.001 au electron density isosurface for (a) pure **14ditfb** and its 1:1 complexes, (b) pure **13ditfb** and its 1:1 complexes, (c) pure **135titfb** and its 1:1 complexes, and (d) pure **135titfb** and its 1:2 complexes. Black curves represent the pure donors, while the colored curves represent halogen-bonded complexes with corresponding acceptor (see legend).

The reduction of ESP_max_ (and the energies of binding of subsequent molecules) is in accord with the observed high occurrence of crystal structures where the donors do not achieve their maximal topicity. Another question requiring attention is the effect of the basicity of the acceptor on the topicity of the donor. As demonstrated by the crystal structures of cocrystals with **135titfb** with simple heterocyclic acceptors, there is a definite increase in the probability of achieving higher topicities with the increase of the basicity of the acceptor. However, one would also expect stronger bases to exact a stronger influence on the donor molecule and to reduce the ESP_max_ on free iodine atoms (and subsequently the binding energy for subsequent molecules) more than the weaker ones. In order to elucidate this issue, we have performed additional computations with two extremes, 4-cyanopyridine and 4-(*N*,*N*-dimethylamino)pyridine, binding to **135titfb**. To provide a more detailed view of the changes of the ESP with binding of base molecules, we have plotted ESP as the function of angle φ (Scheme 2) which corresponds to the deflection from linearity (XB angle −180°) in the plane of the donor molecule (Figure 6). The maxima of the obtained curves occur at φ = 0 and correspond to the ESP_max_ of the σ-hole, while the values of φ where ESP(φ) changes sign (ESP(φ_max_) = 0) indicate the angular width of the σ-hole (i.e., the ESP(φ) is positive in the region −φ_max_ > φ > φ_max_).

As can be observed, the ESP(φ) is globaly lowered upon binding of the base molecules (however maintaining the shape), leading to the reduction of both ESP_max_ and φ_max_. As expected, the effect increases with the basicity of the base—binding of **4cnpy**, **py**, and **dmap** reduces ESP_max_ by 10.7, 22.0, and 31.5 kJ mol^–1^ e^–1^ and φ_max_ by 4.5°, 10.5°, and 14.5°, respectively. Also, the effect appears to be nearly proportional to the number of bound acceptor molecules—upon binding of two acceptor molecules, ESP_max_ on the third iodine atom is reduced by 20.0, 41.3, and 58.3 kJ mol^–1^ e^–1^, while φ_max_ is reduced by 9.0°, 18.1°, and 24.4°. As can be seen, this effect can be (depending of the basicity and the number of acceptor molecules) significant—specifically, the binding of two **dmap** molecules on **135titfb** reduces ESP_max_ on the third iodine atom by 46%, making it a much weaker Lewis acid as compared to free **135titfb**.

**Figure 7:**
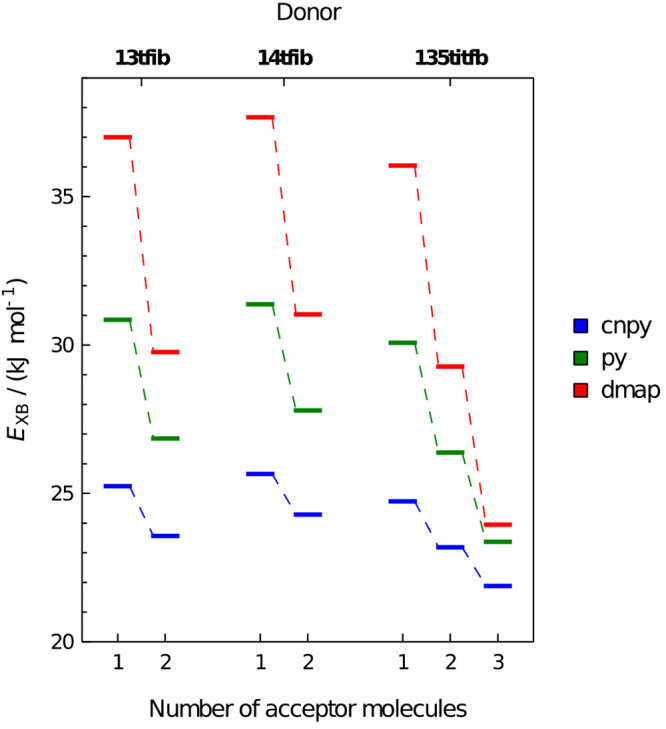
First, second, and (for the case of **135titfb**) third binding energy of **dmap**, **py**, and **4cnpy** to donors **14ditfb**, **13ditfb**, and **135titfb**.

This result is apparently in contrast with the experimental observation that only the strongest bases (**dmap** in particularly) form 1:3 cocrystals, whereas the weakest bases (such as **4cnpy**) often form 1:1 cocrystals. One should keep in mind, however, that the halogen bond energy is also highly influenced by the basicity of the acceptor: the difference in XB energies formed by *N*-halogenosuccinimides (halogen = chlorine, bromine, and iodine) with **dmap** and **4cnpy** was found to be as much as 20 kJ mol^–1^.^25^ The differences between XB energies for binding of the first base molecule to **135titfb** as the XB donor are somewhat less—36.0 kJ mol^–1^ for **dmap** and 24.7 kJ mol^–1^ for **4cnpy** with an intermediary energy of 30.1 kJ mol^–1^ for **py**, it being an intermediary base (Figure 7). As noted above, the energy of binding the second molecule of **py** is by 3.7 kJ mol^–1^, and the third is by ca. 6.7 kJ mol^–1^ less than the first one. Upon binding of **dmap**, the reduction of binding energy is considerably more pronounced (Δ*E* of 6.8 kJ mol^–1^ and 12.1 kJ mol^–1^), while with **4cnpy** it is considerably less (1.6 kJ mol^–1^ and 2.9 kJ mol^–1^), again mirroring the differences in the reduction of ESP_max_ upon binding of each of the three bases. However, in spite of the largest reduction of binding energies upon binding of subsequent molecules of **dmap**, the overall binding energies of **dmap** are still considerably higher than those of weaker bases. Indeed, the binding energy of the third molecule of **dmap** to **135titfb** (23.9 kJ mol^–1^) is higher than the binding energy of the second molecule of **4cnpy** (23.2 kJ mol^–1^). Therefore, although the reduction of the Lewis acidity of the free iodine atom(s) of the **135titfb** molecule is most pronounced with the strongest bases as acceptors, the increase of halogen bond energy due to the basicity of the acceptor more than compensates its decrease due to the reduction of the acidity of the donor.

While the p*K*_a_-dependent topicity of **135titfb** can be explained by the above considerations, the disparate behavior of the other two donors—the generally monotopic **13ditfb** and generally ditopic **14ditfb**—does not seem to stem from similar reasons. Although the ESP_max_ of **14ditfb** is somewhat higher than that of **13ditfb**, its reduction upon binding a single base molecule is smaller and the energies of binding of both base molecules are higher (for all three studied bases, Figure 7); these differences are minute and do not seem to be able to account for the observed difference in behavior. The binding energy of the second molecule of an intermediate base (**py**) is for both donors considerably higher than the energy of binding the first molecule of the weak base (**4cnpy**). Therefore, as both XB donors can form the 1:1 cocrystal with **4cnpy**, they should both be expected to form 1:2 cocrystals with intermediate (and even more so with strong) bases. It is therefore probable that the main determining factor for the topicity of **13ditfb** and **14ditfb** is not the energy of the halogen bonds they form, but rather the ability of the resulting complexes to achieve close packing. When the linear **14ditfb** binds a pair of base molecules, the resulting complex is also linear, and generally centrosymmetric, and therefore likely to efficiently fill the space in a crystal structure. The bent 2:1 complex resulting from binding two base molecules on **13ditfb** is sterically more demanding, and less likely to be able to achieve close packing.

## Conclusions

The structural and statistical study have pointed out significant differences in topicities of the **13ditfb** (monotopic) and **14ditfb** (ditopic) molecules as halogen bond donors in cocrystals with monotopic pyridines. It has been shown that the preferential cocrystal stoichiometry (and donor topicity) for both donor molecules is generally independent of the acceptor basicity but is rather determined by the overall crystal structure. Thus, **13ditfb** will be the predominantly ditopic donor in cocrystals with polytopic acceptors, while the bent 2:1 complexes it forms with monotopic bases only exceptionally can efficiently pack in a crystal structure, resulting in the predominance of 1:1 cocrystals.

The topicity of **135titfb** in cocrystals with monotopic pyridines highly depends on the basicity of the used acceptors—in combinations with more basic pyridines, **135titfb** forms three halogen bonds, but with others, one or two bonds have formed. DFT calculations have shown that binding of the one acceptor molecule to the accessible iodine atoms leads to the reduction of the ESP_max_ values on free iodine atoms, and this effect increases with the basicity of the base. However, stronger bases form stronger halogen bonds, and the corresponding increase in bond energy can compensate the reduction of the Lewis acidity of the donors, thus allowing the formation of the three halogen bonds. Obtained results indicate that in addition to the donor molecule itself, the number of halogen bonds in the crystal structure is predetermined by the topicity and basicity of the acceptor molecule, which have been recognized as important factors in both the formation of the halogen-bonded molecular complexes and their packing in the solid state.

## Experimental Section

### Synthesis of Cocrystals

All the solvents and compounds used as halogen bond acceptors were procured from Sigma-Aldrich Chemie GmbH, Taufkirchen, Germany, and used without additional purification. Halogen bond donors **13tfib**, **14tfib**, and **135titfb** were procured from Manchester Organics Ltd., Cheshire, UK and used without additional purification.

Cocrystals of **14ditfb** and **135titfb** and acceptors used have been prepared by both grinding and crystallization from solution. The grinding experiments were conducted in a Retsch MM200 ball mill using 10 mL stainless steel jars and two stainless steel balls (5 mm in diameter) for 15 min, under normal laboratory condition (40–60% relative humidity and temperature ca. 25 °C). Due to the large number of experiments performed, masses and volumes of the reactants used in the mechanochemical synthesis of cocrystals are given in Tables S5 and S6 in the Supporting Information. Single crystals of cocrystals with liquid acceptors were prepared by dissolving a halogen bond donor **135titfb** or **14ditfb** (50 mg) in hot ethanol (1.5 mL), after which a large excess of liquid acceptor was added (500 μL). The resulting solution was stirred and left at room temperature. Single crystals of **135tfib** cocrystal with **4cnpy** were prepared by dissolving donor *m*(**135titfb**) = 48 mg and acceptor *m*(**4cnpy**) = 53 mg in 1.00 mL of hot ethanol, after which solution was left at room temperature.

Cocrystals of **13ditfb** and solid acceptors (**acr**, **4cnpy**, and **dmap**) have been prepared by grinding as described above, while the cocrystals with liquid acceptors were synthesized by mixing of **13ditfb** and corresponding acceptor in a 1:2 stoichiometric ratio on a microscope glass slide, after which crystallization of the product occurred. Masses and volumes of the reactants used in the synthesis of cocrystals are given in Table S7 in the Supporting Information. Single crystals of **13ditfb** cocrystals were prepared by dissolving a halogen bond donor (40 μL) in hot ethanol (1.5 mL), after which a large excess of corresponding acceptor was added (500 μL).

### X-ray Diffraction Experiments

Single crystal X-ray diffraction experiments were performed using an Oxford Diffraction Xcalibur Kappa CCD X-ray diffractometer with graphite-monochromated Mo Kα (λ = 0.71073 Å) radiation. The data sets were collected using the ω-scan mode over the 2θ range up to 54°. Programs CrysAlis CCD and CrysAlis RED were employed for data collection, cell refinement, and data reduction.^46,47^ The structures were solved by direct methods and refined using the SHELXS and SHELXL programs, respectively.^48,49^ The structural refinement was performed on *F*^2^ using all data. The hydrogen atoms were placed in calculated positions and treated as riding on their parent atoms [C–H = 0.93 Å and *U*_iso_(H) = 1.2 *U*_eq_(C); C–H = 0.97 Å and *U*_iso_(H) = 1.2 *U*_eq_(C)]. All calculations were performed using the WinGX crystallographic suite of programs.^50^ The figures were prepared using Mercury.^51^

Powder X-ray diffraction experiments on the samples were performed on an Aeris X-ray diffractometer (Malvern Panalytical, Malvern Worcestershire, UK) with Cu Kα_1_ (λ = 1.54056 Å) radiation. The scattered intensities were measured with a PIXcel-1D-Medipix3 detector. The angular range was from 5° to 40° (2θ) with a continuous step size of 0.02° and measuring a time of 0.5 s per step. Data collection methods were created using the program package START XRDMP CREATOR (Malvern Panalytical, Malvern Worcestershire, UK), while the data were analyzed using X’Pert HighScore Plus (Version 2.2, Malvern Panalytical, Malvern Worcestershire, UK).^52^

### Thermal Analysis

Differential scanning calorimetry (DSC) and thermogravimetric (TG) measurements were performed simultaneously on a Mettler-Toledo TGA/DSC 3+ module (Mettler Toledo, Greifensee, Switzerland). Samples were placed in alumina crucibles (40 μL) and heated 25 to 300 °C, at a heating rate of 10 °C min^–1^ under nitrogen flow of 150 mL min^–1^.

Data collection and analysis were performed using the program package STARe Software (Version 15.00, Mettler Toledo, Greifensee, Switzerland).^53^ TG and DSC thermograms of the prepared compounds are shown in Figures S11–S15 in Supporting Information.

### Calculation Details

All calculations were performed with Gaussian 09 (Rev. D.01) program suite.^54^ Geometry optimization of all donors, acceptors, and halogen-bonded complexes has been performed using B3LYP functional^55^ with Grimme’s GD3 dispersion correction^56^ and def2-TZVP basis set^57^ with effective core potential (ECP) for iodine atoms. Parameters of basis set and ECP for iodine were taken from the EMSL website.^58^ The same level of theory was used to calculate the binding energies on optimized geometries, employing the Boys–Bernardi counterpoise scheme^59^ to account for basis set superposition error and neglecting the relaxation energies of monomers, due to the high rigidity of all donors and acceptors. For overall population analysis (electron density, electrostatic potential and NBO analysis), single-point calculations on CAM-B3LYP^60^/def2-QZVP (with ECP for I) level were performed on optimized geometries.

Electron density and electrostatic potential were computed in a box around the halogen atom with length of 12.0 au using the *cubegen* utility of Gaussian. The number of points used was 150 in each dimension in the plane of the halogen bond donor molecule. Obtained densities were fitted with linear regression using basis of the type:
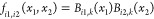
where *B*_*i*,*k*_(*x*) is the *i*th *B*-spline function of the order *k*. *B*-spline functions were defined on the same box, and the order used was *k* = 4. The number of the B-spline function used was 30 in each dimension, so that there are 25 data points per coefficient that are fitted. An analogous procedure was used to fit the electrostatic potential in the plane of the donor molecule.

Fitted electron density was used to compute the isodensity curve in the plane of interest. This was done by defining a line in the plane which is perpendicular to the halogen bond and taking 1000 equidistant points along it. From each point, a linear search was performed in the direction perpendicular to the line (and parallel to the halogen bond) until the point with the desired density was found. For each point found, the value of electrostatic density was computed from the fit.

### Database Survey

A data survey has been performed on the CSD database, version 5.42 (May 2021) with three updates using ConQuest Version 2020.3.0. For the halogen-bonded contacts, the upper limit of the distance between the donor atom (iodine) and the acceptors was defined as the sum of their van der Waals radii. In order to ascertain the frequency of halogen bonding, for each donor a number of searches were made: search for the total number of structures including perfluorinated halobenzenes as halogen bond donors; search which included the structures of the corresponding donor and nitrogen-containing molecule (defined as “N” fragment in ConQuest) which either can or cannot participate in halogen bonding; search for the structures in which donor and nitrogen-containing molecule participate in one or more I···N halogen bonds; search for structures of perfluorinated halobenzenes and pyridine derivatives from which structures with mono- and polytopic pyridines participating in one or more halogen bonds are manually extracted.

## Supporting Information Available

ORTEP representations of formula units, PXRD patterns, DSC curves, crystallographic data, synthetic details and crystal structure descriptions (PDF)

## Accession Codes

CCDC 2128222–2128241 contain the supplementary crystallographic data for this paper. These data can be obtained free of charge via www.ccdc.cam.ac.uk/data_request/cif, or by emailing data_request@ccdc.cam.ac.uk, or by contacting The Cambridge Crystallographic Data Centre, 12 Union Road, Cambridge CB2 1EZ, UK; fax: +44 1223 336033.
