# Supplementary material for: Anticooperativity of Multiple Halogen Bonds and Its Effect on Stoichiometry of Cocrystals of Perfluorinated Iodobenzenes
Source: Cryst Growth Des. 2022 Mar 24;22(4):2644–53. doi: 10.1021/acs.cgd.2c00077 (PMC8991082; doi:10.1021/acs.cgd.2c00077)
Supplement: Supplementary file 1 — cg2c00077_si_001.pdf [file cg2c00077_si_001.pdf]

Anticooperativity of multiple halogen bonds and its effect on stoichiometry of cocrystals of perfluorinated iodobenzenes

*Nikola Bedeković[1], Tomislav Piteša[2], Mihael Eraković[2], Vladimir Stilinović[*1], Dominik Cinčić[*1]*

[1]Department of Chemistry, Faculty of Science, University of Zagreb, Horvatovac 102a Zagreb, Croatia.

[2] Ruđer Bošković Institute, Bijenička cesta 54, 10 000 Zagreb

E-mail: dominik@chem.pmf.hr, vstilinovic@chem.pmf.hr

SUPPORTING INFORMATION

Table of contents

Crystallographic data of the prepared compounds (pages 2–6)

ORTEP plots of an asymmetric units of the prepared compounds (pages 7–13)

Crystal structure descriptions and halogen bond parameters (pages 13–18)

Masses and volumes of the reactants used in cocrystal synthesis (pages 19–21)

XRPD patterns of the prepared compounds (pages 22–23)

DSC curves of the prepared compounds (pages 24–37)

Table S1. Crystallographic data of the prepared compounds.

	**(13tfib)(24lut)**	**(13tfib)(26lut)**	**(13tfib)(34lut)**	**(14tfib)(24lut)**$_2$
Molecular formula	$C_{13}H_9NF_4I_2$	$C_{13}H_9NF_4I_2$	$C_{13}H_9NF_4I_2$	$C_{20}H_{18}N_2F_4I_2$
M / g mol^{-1}	509.4	509.4	509.4	616.0
Crystal system	monoclinic	monoclinic	monoclinic	triclinic
Space group	$P2_1/c$	$C2/c$	$P2_1/c$	$P\bar{1}$
a / Å	9.0971(3)	17.2748(62)	8.6165(17)	7.3917(7)
b/ Å	7.6003(3)	15.0698(22)	22.5549(34)	8.7189(9)
c/ Å	21.8670(7)	14.9191(42)	8.1997(15)	12.1289(13)
α	90	90	90	74.410(9)
β	100.524(4)	128.763(50)	114.054(23)	78.525(9)
γ	90	90	90	81.642(9)
V / Å^3	1486.47(11)	3028.41(10)	1455.18(164)	734.36(23)
Z	2	8	4	2
T / K	150	150	150	150
ρ_{calc} / g cm^{-3}	2.27	2.23	2.32	2.30
μ (Mo-K_α) / mm^{-1}	4.263	4.185	4.354	4.314
$\theta_{min,max}$	$3{,}9 \leq \theta \leq 27.0$	$3{,}9 \leq \theta \leq 27.0$	2.7	$3{,}9 \leq \theta \leq 25.0$
F(000)	944.0	1888.0	27.0	472.0
Refl. measured	7266	4636	21218	3128
Refl. unique	3223	2701	3170	2431
Refl. Obs.	2752	1836	2925	1763
R_{int}	0.020	0.031	0.035	0.036
No. parameters	183	183	181	183
$R[F^2 > 2\sigma F^2]$	0.020	0.050	0.056	0.065
$wR(F^2)$	0.045	0.134	0.020	0.182
S	1.012	0.947	1.107	1.005
$\Delta\rho_{max}$ / e Å^{-3}	0.511	2.215	0.818	3.415
$\Delta\rho_{min}$ / e Å^{-3}	−0.493	−1.430	-0.631	−2.650

Continuation of the Table S1.

	**(14tfib)(34lut)$_2$**	**(14tfib)(3acp)**	**(14tfib)(4acp)$_2$**	**(135titfb)(2pic)$_3$**
Molecular formula	$C_{20}H_{18}N_2F_4I_2$	$C_{13}H_7NOF_4I_2$	$C_{24}H_{14}N_2F_4I_2$	$C_{24}H_{28}N_3F_3I_3$
M / g mol^{-1}	616.0	523.01	644.1	523.01
Crystal system	triclinic	triclinic	triclinic	orthorhombic
Space group	$P\bar{1}$	$P\bar{1}$	$P\bar{1}$	$Aba2$
a / Å	6.4742(5)	5.9294(3)	6.2642(3)	7.4381(4)
b/ Å	9.3191(6)	8.9540(6)	8.7611(4)	24.6983(12)
c/ Å	9.3601(6)	10.7523(8)	10.5903(6)	14.6683(8)
α	73.836(5)	70.963(6)	109.850(5)	90
β	79.755(5)	81.150(5)	101.381(5)	90
γ	72.212(6)	76.701(5)	100735(4)	90
V / Å^3	513.81(12)	523.236(1)	515.31(16)	2694.69
Z	2	2	2	4
T / K	150	150	150	150
ρ_{calc} / g cm^{-3}	1.99	2.31	2.08	1.93
μ (Mo-K_α) / mm^{-1}	3.104	4.222	3.107	3.516
$\theta_{min,max}$	$4,2 \leq \theta \leq 27.0$	$4,2 \leq \theta \leq 27.0$	$4,3 \leq \theta \leq 27,0$	3.2
F(000)	294.0	242.0	306.0	27.0
Refl. measured	1953	2976	3863	11499
Refl. unique	1678	2678	2206	2866
Refl. Obs.	1555	2415	2037	2524
R_{int}	0.033	0.029	0.061	0.070
No. parameters	129	190	138	175
$R[F^2 > 2\sigma F^2]$	0.044	0.062	0.037	0.063
$wR(F^2)$	0.117	0.176	0.092	0.146
S	1.014	1.060	1.059	1.071
$\Delta\rho_{max}$ / e Å^{-3}	1.658	1.469	1.439	3.940
$\Delta\rho_{min}$ / e Å^{-3}	−2.463	−1.969	−2.236	-3.014

Continuation of the Table S1.

	(135tftib)(3pic)$_3$	(135tftib)(24lut)$_2$	(135tftib)(34lut)$_3$	(135tftib)(35lut)$_3$
Molecular formula	$C_{24}H_{28}N_3F_3I_3$	$C_{20}H_{18}N_2F_3I_3$	$C_{27}H_{27}N_3F_3I_3$	$C_{27}H_{27}N_3F_3I_3$
M / g mol^{-1}	523.01	724.1	831.2	831.2
Crystal system	monoclinic	triclinic	monoclinic	orthorhombic
Space group	$P2_1/c$	$P\bar{1}$	$P2_1$	$Aba2$
a / Å	7.4670(6)	8.8100(7)	7.2806(20)	7.3190(11)
b/ Å	15.2254(10)	9.0632(7)	25.8013(50)	26.5945(26)
c/ Å	23.0047(18)	14.8394(11)	8.1438(20)	15.2056(10)
α	90	84.770(6)	90	90
β	92.897(7)	75.787(7)	105.176(30)	90
γ	90	84.101(6)	90	90
V / Å^3	2612.02(10)	1139.85(22)	1476.45(125)	2959.70(29)
Z	4	2	2	4
T / K	150	150	150	150
ρ_{calc} / g cm^{-3}	2.01	2.11	1.81	1.87
μ (Mo-K_α) / mm^{-1}	3.628	4.145	3.211	3.207
$\theta_{min,max}$	$3,8 \leq \theta \leq 27.0$	$4,1 \leq \theta \leq 27.0$	$3,7 \leq \theta \leq 27,0$	$4,1 \leq \theta \leq 27.0$
F(000)	1488.0	676.0	762.0	1584.0
Refl. measured	12316	7811	15771	6686
Refl. unique	5614	4828	3281	3034
Refl. Obs.	3568	3735	2327	2361
R_{int}	0.055	0.047	0.057	0.032
No. parameters	301	257	190	169
$R[F^2 > 2\sigma F^2]$	0.046	0.052	0.042	0.027
$wR(F^2)$	0.098	0.138	0.112	0.064
S	0.866	1.021	0.896	0.896
$\Delta\rho_{max}$ / e Å^{-3}	2.261	2.104	1.393	0.461
$\Delta\rho_{min}$ / e Å^{-3}	−1.449	−1.993	−0.695	−0.662

Continuation of the Table S1.

	**(135tftib)(246kol)$_3$**	**(135tftib)(3acp)**	**(135tftib)(4acp)$_2$**	**(135tftib)(qin)**
Molecular formula	$C_{30}H_{33}N_3F_3I_3$	$C_{13}H_7NF_3I_3$	C20H14$N_2O_2F_3I_3$	$C_{15}H_7NF_3I_3$
M / g mol^{-1}	873.3	630.9	752.05	638.9
Crystal system	monoclinic	monoclinic	orthorhombic	orthorhombic
Space group	$C2/c$	$P2_1/c$	$C2/c$	$Pbca$
a / Å	28.413(3)	15.0460(10)	32.818(3)	17.9016(13)
b/ Å	16.388(2)	8.2903(4)	9.2579(10)	7.3573(6)
c/ Å	22.645(3)	14.4172(8)	7.5254(8)	25.2611(17)
α	90	90	90	90
β	111.510(15)	111.176(7)	90.10(2)	90
γ	90	90	90	90
V / Å^3	9809.87(4)	1676.91(49)	2286.41(4)	3327.07(4)
Z	12	4	4	8
T / K	150	150	150	150
ρ_{calc} / g cm^{-3}	1.78	2.50	2.18	2.55
μ (Mo-K_α) / mm^{-1}	2.918	5.618	4.144	5.660
$\theta_{min,max}$	$3,8 \leq \theta \leq 27.0$	$3,9 \leq \theta \leq 27.0$	$4,4 \leq \theta \leq 25.0$	$3,8 \leq \theta \leq 27,0$
F(000)	5040.0	1144.0	1400.0	2320.0
Refl. measured	229999	6185	12120	21807
Refl. unique	10513	3575	1980	3616
Refl. Obs.	4686	2651	1764	2471
R_{int}	0.085	0.027	0.086	0.083
No. parameters	545	191	139	199
$R[F^2 > 2\sigma F^2]$	0.047	0.030	0.032	0.041
$wR(F^2)$	0.092	0.067	0.068	0.079
S	0.728	0.895	1.047	0.907
$\Delta\rho_{max}$ / e Å^{-3}	1.143	0.856	0.832	1.624
$\Delta\rho_{min}$ / e Å^{-3}	−1.454	−0.980	−0.815	−1.408

Continuation of the Table S1.

	**(135tftib)(iqin)$_2$**	**(135tftib)(4cnp)**	**(135tftib)(akr)**
Molecular formula	$C_{24}H_{14}N_2F_3I_3$	$C_{12}H_4N_2F_3I_3$	$C_{19}H_9NF_3I_3$
M / g mol^{-1}	768.1	629.88	688.9
Crystal system	triclinic	monoclinic	monoclinic
Space group	$P\bar{1}$	$P2_1/n$	$P2_1/c$
a / Å	7.9790(4)	4.2374(5)	10.4746(4)
b/ Å	8.8600(4)	14.2555(20)	12.5092(4)
c/ Å	17.6834(7)	26.5477(28)	15.1548(5)
α	89.789(3)	90	90
β	81.841(4)	93.351(10)	102.576(3)
γ	73.975(4)	90	90
V / Å^3	1188.54(16)	1600.91(10)	1938.08(13)
Z	2	4	4
T / K	150	150	150
ρ_{calc} / g cm^{-3}	2.15	2.55	2.36
μ (Mo-K_α) / mm^{-1}	3.983	5.878	4.868
$\theta_{min,max}$	$4,0 \leq \theta \leq 27.0$	$4,1 \leq \theta \leq 27.0$	$3,7 \leq \theta \leq 27,0$
F(000)	716.0	1104.0	1264.0
Refl. measured	8125	6684	11261
Refl. unique	5097	3408	4215
Refl. Obs.	4094	2477	2535
R_{int}	0.030	0.036	0.054
No. parameters	289	181	235
$R[F^2 > 2\sigma F^2]$	0.033	0.036	0.032
$wR(F^2)$	0.078	0.077	0.057
S	0.954	0.889	0.683
$\Delta\rho_{max}$ / e Å^{-3}	1.138	1.904	0.713
$\Delta\rho_{min}$ / e Å^{-3}	−1.498	−1.381	−0.824

ORTEP plots of an asymmetric units of the prepared compounds

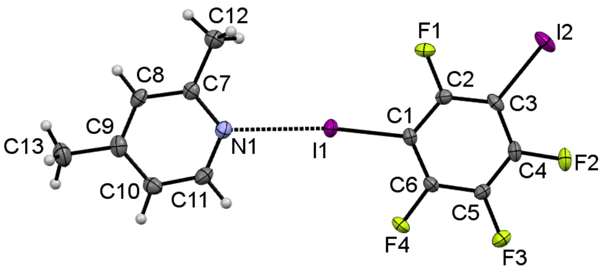

Figure S1. Molecular structure of (**13tfib**)(**24lut**) showing the atom-labelling scheme. Displacement ellipsoids are drawn at the 50 % probability level, and H atoms are shown as small spheres of arbitrary radius.

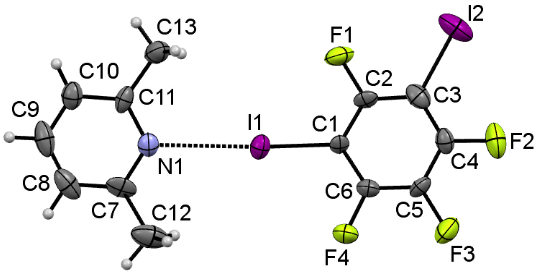

Figure S2. Molecular structure of (**13tfib**)(**26lut**) showing the atom-labelling scheme. Displacement ellipsoids are drawn at the 50 % probability level, and H atoms are shown as small spheres of arbitrary radius.

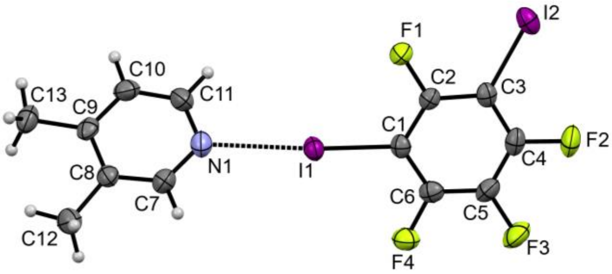

Figure S3. Molecular structure of (**13tfib**)(**34lut**) showing the atom-labelling scheme. Displacement ellipsoids are drawn at the 50 % probability level, and H atoms are shown as small spheres of arbitrary radius.

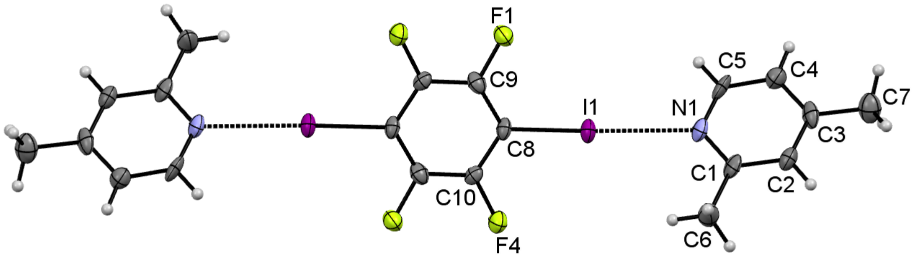

Figure S4. Molecular structure of (**14tfib**)(**24lut**) showing the atom-labelling scheme. Displacement ellipsoids are drawn at the 50 % probability level, and H atoms are shown as small spheres of arbitrary radius.

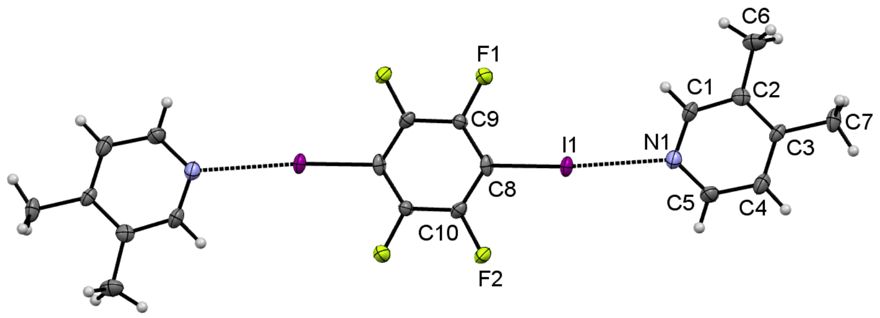

Figure S5. Molecular structure of (**14tfib**)(**34lut**) showing the atom-labelling scheme. Displacement ellipsoids are drawn at the 50 % probability level, and H atoms are shown as small spheres of arbitrary radius.

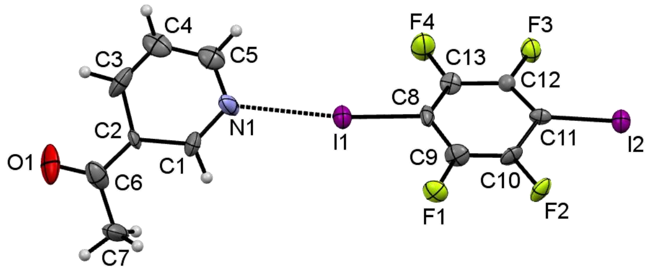

Figure S6. Molecular structure of (**14tfib**)(**3acpy**) showing the atom-labelling scheme. Displacement ellipsoids are drawn at the 50 % probability level, and H atoms are shown as small spheres of arbitrary radius.

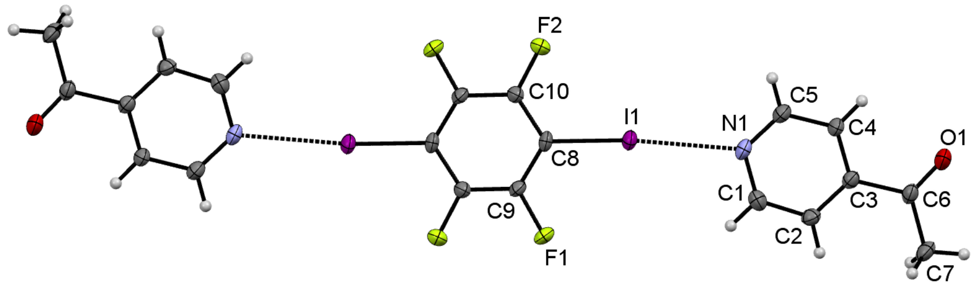

Figure S7. Molecular structure of (**14tfib**)(**4acpy**) showing the atom-labelling scheme. Displacement ellipsoids are drawn at the 50 % probability level, and H atoms are shown as small spheres of arbitrary radius.

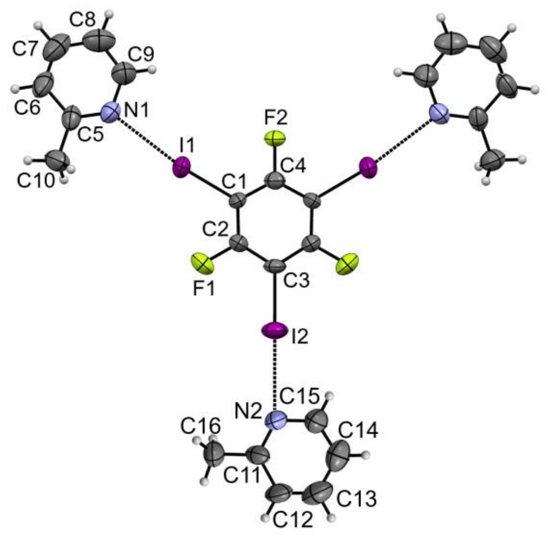

Figure S8. Molecular structure of (**135tftib**)(**2pic**)$_3$ showing the atom-labelling scheme. Displacement ellipsoids are drawn at the 50 % probability level, and H atoms are shown as small spheres of arbitrary radius.

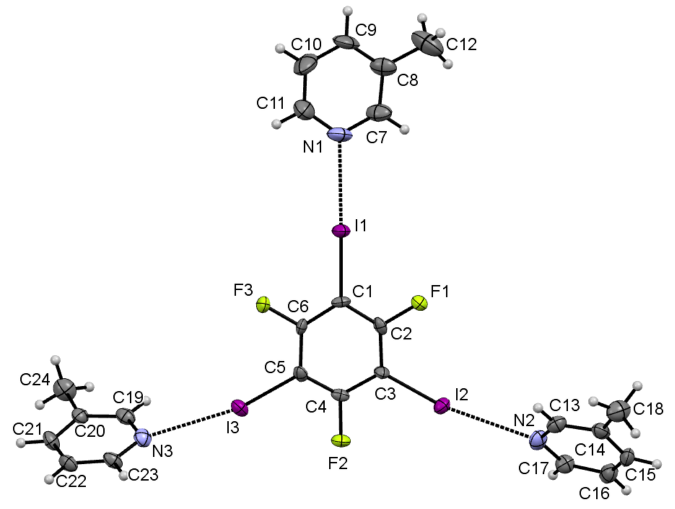

Figure S9. Molecular structure of (**135tftib**)(**3pic**)$_3$ showing the atom-labelling scheme. Displacement ellipsoids are drawn at the 50 % probability level, and H atoms are shown as small spheres of arbitrary radius.

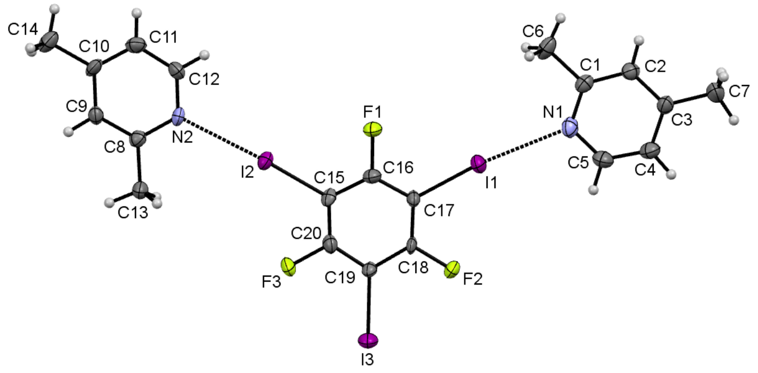

Figure S10. Molecular structure of (**135tftib**)(**24lut**)$_2$ showing the atom-labelling scheme. Displacement ellipsoids are drawn at the 50 % probability level, and H atoms are shown as small spheres of arbitrary radius.

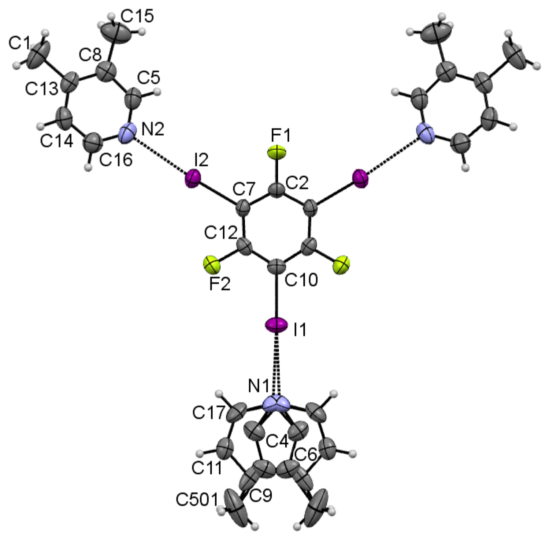

Figure S11. Molecular structure of (**135tftib**)(**34lut**)$_3$ showing the atom-labelling scheme. Displacement ellipsoids are drawn at the 50 % probability level, and H atoms are shown as small spheres of arbitrary radius.

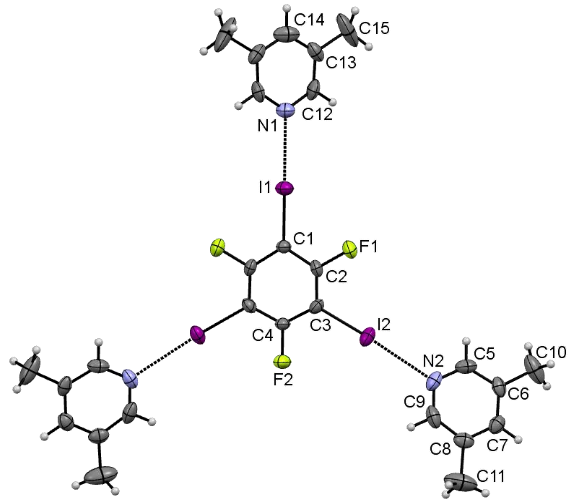

Figure S12. Molecular structure of (**135tftib**)(**35lut**)$_3$ showing the atom-labelling scheme. Displacement ellipsoids are drawn at the 50 % probability level, and H atoms are shown as small spheres of arbitrary radius.

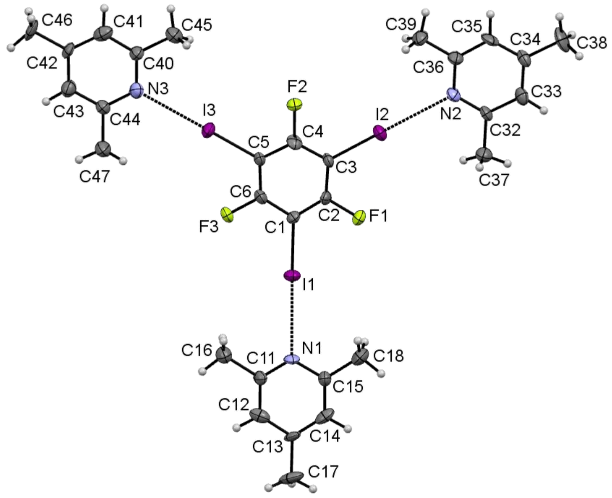

Figure S13. Molecular structure of (**135tftib**)(**246col**)$_3$ showing the atom-labelling scheme. Displacement ellipsoids are drawn at the 50 % probability level, and H atoms are shown as small spheres of arbitrary radius.

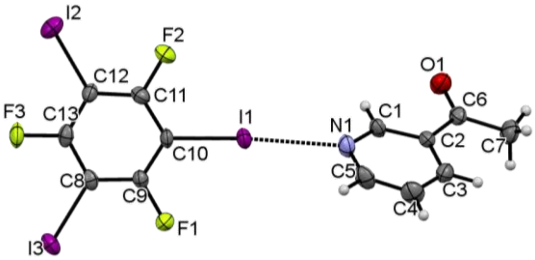

Figure S14. Molecular structure of (**135tftib**)(**3acpy**) showing the atom-labelling scheme. Displacement ellipsoids are drawn at the 50 % probability level, and H atoms are shown as small spheres of arbitrary radius.

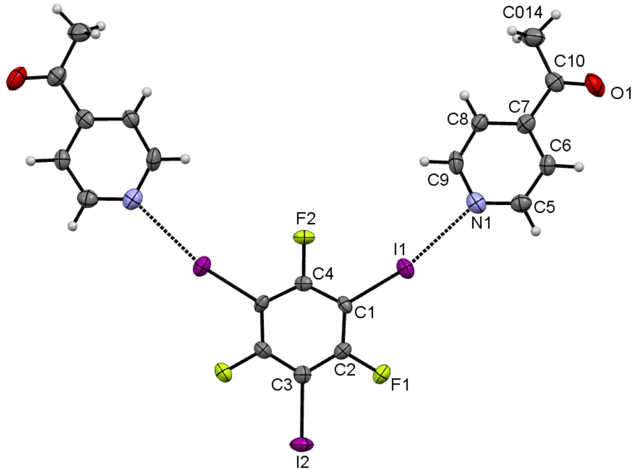

Figure S15. Molecular structure of (**135tftib**)(**4acpy**)$_2$ showing the atom-labelling scheme. Displacement ellipsoids are drawn at the 50 % probability level, and H atoms are shown as small spheres of arbitrary radius.

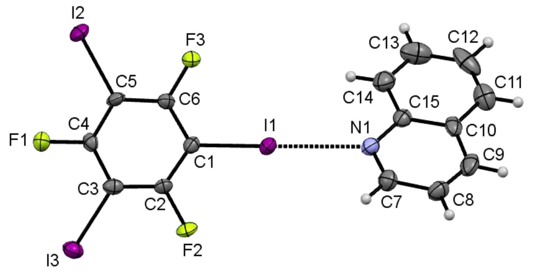

Figure S16. Molecular structure of (**135tftib**)(**qin**) showing the atom-labelling scheme. Displacement ellipsoids are drawn at the 50 % probability level, and H atoms are shown as small spheres of arbitrary radius.

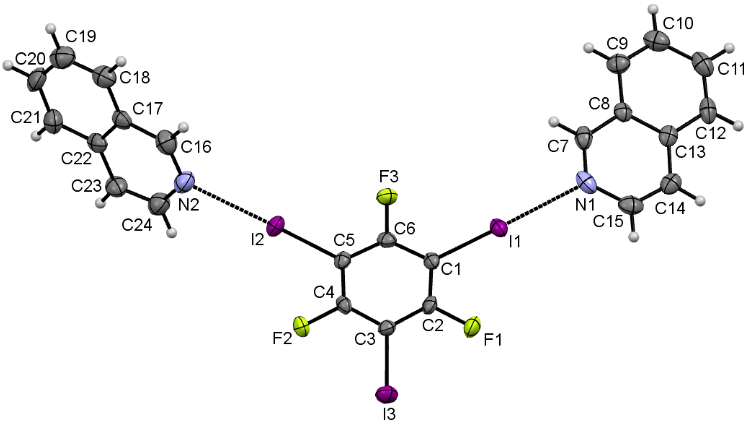

Figure S17. Molecular structure of (**135tftib**)(**iqin**)$_2$ showing the atom-labelling scheme. Displacement ellipsoids are drawn at the 50 % probability level, and H atoms are shown as small spheres of arbitrary radius.

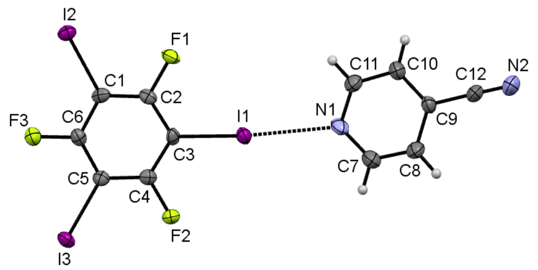

Figure S18. Molecular structure of (**135tftib**)(**4cnpy**) showing the atom-labelling scheme. Displacement ellipsoids are drawn at the 50 % probability level, and H atoms are shown as small spheres of arbitrary radius.

Crystal structures of the prepared cocrystals

Crystal structures of the **13tfib** cocrystals

Starting from **13tfib** as donor and **24lut**, **26lut** and **34lut** as XB acceptors, three coocrystals of 1:1 stoichiometry have formed (Fig S19). One halogen bond between pyridine nitrogen and iodine atom has formed in all three compounds, while second iodine is bonded to fluorine– (**13tfib**)(**24lut**), (**13tfib**)(**26lut**) or iodine atom – (**13tfib**)(**34lut**) of an adjacent donor molecules. Formed halogen bond is the shortest in (**13tfib**)(**34lut**) (d(I···N)= 2.697(2) Å) and followed by (**13tfib**)(**24lut**) (d(I···N) = 2.815(3) Å) and (**13tfib**)(**26lut**) (d(I···N) = 2.948(6) Å). Such distances can be explained in terms of more sterical hindrance between **13tfib** molecule and acceptors with methyl group in ortho position (**24lut** and **26lut**), resulting in increase of the bond length.

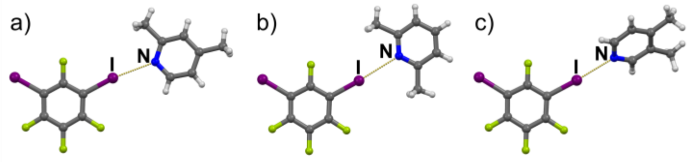

Figure S19. Halogen bonded complexes in crystal structures of a) (**13tfib**)(**24lut**), b) (**13tfib**)(**26lut**) and c) (**13tfib**)(**34lut**).

Table S2. Geometrical parameters of halogen bonds in **13tfib** cocrystals.

compound	halogen bond	*d* / Å	*r.s.* / %	ϑ(C-I···N)/°
(**13titfb**)(**4cnpy**)	I1···N1	3.001(1)	15.0	173.5(4)
(**13titfb**)(**qin**)	I1···N1	2.895(6)	18.0	173.2(2)
	I2···N2	2.912(7)	17.5	170.3(2)
(**13titfb**)(**iqin**)	I1···N1	2.842(6)	19.5	176.8(2)
(**13titfb**)(**acr**)	I1···N1	2.992(3)	15.2	176.3(1)
(**13titfb**)(**35lut**)	I1···N1	2.847(5)	19.3	175.5(2)
(**13titfb**)(**34lut**)	I1···N1	2.697(2)	23.6	175.3(1)
(**13titfb**)(**24lut**)	I1···N1	2.815(3)	20.2	171.5(9)
(**13titfb**)(**26lut**)	I1···N1	2.948(6)	16.5	176.7(3)
(**13titfb**)(**246col**)	I1···N1	2.962(8)	16.1	178.6(4)
(**13titfb**)(**dmap**)	I1···N3	2.800(4)	20.7	174.3(2)
	I2···N7	2.783(4)	21.2	171.1(2)
	I3···N5	2.781(4)	21.2	174.6(1)
	I4···N1	2.744(4)	22.2	171.2(1)

<u>Crystal structures of the **14tfib** cocrystals</u>

Cocrystallization of **3acpy**, **4acpy**, **24lut** and **34lut** with XB donor **14tfib** resulted in formation of a four novel cocrystals of 1:2 stoichiometry in which two I···N_{py} halogen bonds have established. Two halogen bonds in each cocrystal are simetrically equivalent and consequently of the same length and angles. The shortest one has found in (**14tfib**)(**26lut**)$_2$ (*d*(I···N)= 2.800(1) Å) which is followed by (**14tfib**)(**34lut**)$_2$ (*d*(I···N)= 2.835(5) Å), (**14tfib**)(**4acpy**)$_2$ (*d*(I···N)= 2.851(4) Å) and (**14tfib**)(**3acpy**)$_2$ (*d*(I···N)= 2.867(3) Å). Molecules are connected in 3D structure by weak C–H···F contacts.

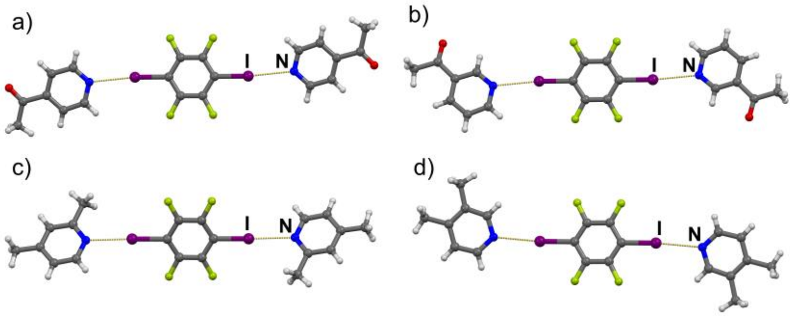

Figure S20. Halogen bonded complexes in crystal structures of a) (**14tfib**)(**4acpy**), b) (**14tfib**)(**3acpy**), c) (**14tfib**)(**24lut**) and d) (**14tfib**)(**34lut**).

Table S3. Geometrical parameters of halogen bonds in **14tfib** cocrystals.

compound	halogen bond	*d* / Å	*r.s.* / %	ϑ(C-I···N)/°
(**14titfb**)(**4cnpy**)	I1···N1	2.946(8)	16.5	172.5(3)
(**14titfb**)(**4acpy**)	I1···N1	2.851(4)	19.2	174.5(1)
(**14titfb**)(**3acpy**)	I1···N1	2.867(3)	18.8	174.9(1)
(**14titfb**)(**qin**)	I1···N1	2.869(3)	18.7	174.6(1)
(**14titfb**)(**iqin**)	I1···N1	2.834(6)	19.7	175.3(2)
(**14titfb**)(**acr**)	I1···N1	2.971(2)	15.8	176.6(8)
(**14titfb**)(**2pic**)	I1···N1	2.871(4)	18.7	175.3(2)
(**14titfb**)(**3pic**)	I1···N1	2.781(3)	21.2	174.4(1)
(**14titfb**)(**4pic**)	I1···N1	2.808(5)	20.4	179.4(2)
(**14titfb**)(**35lut**)	I1···N1	2.802(4)	20.6	173.7(1)
(**14titfb**)(**34lut**)	I1···N1	2.835(5)	19.7	175.3(2)
(**14titfb**)(**24lut**)	I1···N1	2.800(1)	20.7	177.6(4)
(**14titfb**)(**246col**)	I1···N1	3.007(2)	14.8	180.0(1)
(**14titfb**)(**dmap**)	I1···N1	2.667(2)	24.4	179.3(7)

Crystal structures of the **135tfib** cocrystals

Compounds prepared starting from **135tfib** and used XB acceptors can be divided in three groups according to the topicity of the donor molecule in corresponding crystal structure (monotopic, ditopic or tritopic). In cocrystals (**135titfb**)(**acr**), (**135titfb**)(**4cnpy**), (**135titfb**)(**kin**) and (**135titfb**)(**3acpy**) donor molecule is monotopic XB donor (one I···N_{py} halogen bond was formed between donor and acceptor molecules which geometries have shown in Table S4). Additional contacts between donor iodine atom and cyano (I···NC) or keto group (I···O) have been found in crystal structures of (**135titfb**)(**4cnpy**) and (**135titfb**)(**3acpy**). Both contacts participate in connecting molecules in 3D crystal structure, but they are noticeably longer than dominant I···N_{py} interactions and most likely formed as a consequence of crystal packing (Table S4). In other two compounds, molecules are connected in 3D structure by weak C–H···F contacts.

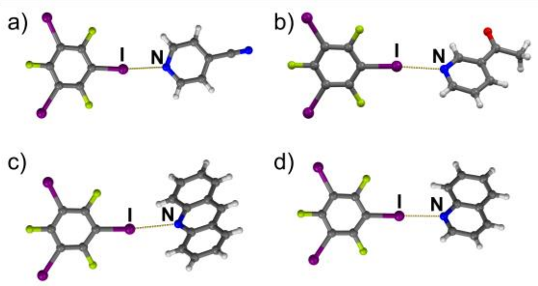

Figure S21. 1:1 halogen bonded complexes in cocrystals a) (**135titfb**)(**4cnpy**), b) (**135titfb**)(**3acpy**) and c) (**135titfb**)(**acr**) and d) (**135titfb**)(**qin**).

Two halogen bonds per donor molecule have been formed in three prepared cocrystals – (**135titfb**)(**ikin**)$_2$, (**135titfb**)(**24lut**)$_2$ and (**135titfb**)(**4acpy**)$_2$. In (**135titfb**)(**4acpy**)$_2$ two halogen bonds are related by simetry and of equal geometries, while in the others a difference is observed both in the XB lengths and angles (Table S4). Interestingly, despite **4acpy** (as well as **3acpy**) contains keto group prone to participate in halogen bonding, such interaction has not been found in crystal structure of (**135titfb**)(**4acpy**)$_2$. Instead, third iodine atom is connected to fluorine atom of the adjacent donor molecule. In other two compounds, crystal packing of halogen-bonded complexes is achieved by weak C–H···F contacts.

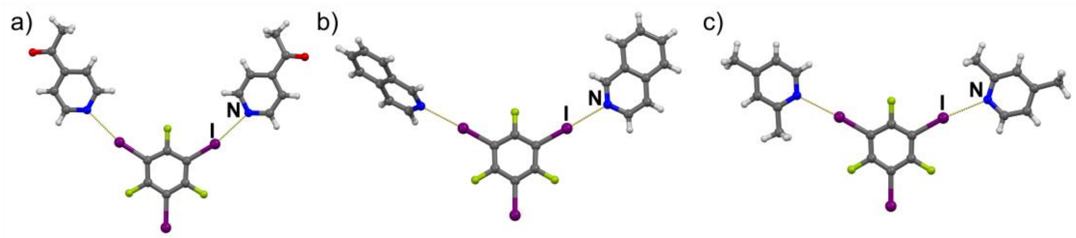

Figure S22. Halogen bonded complexes of the 1:2 stoichiometry in cocrystals a) (**135titfb**)(**iqin**)$_2$, b) (**135titfb**)(**24lut**)$_2$ and c) (**135titfb**)(**4acpy**)$_2$.

Third group of structures are ones in which all donor atoms of **135tfib** participate in halogen bonding with pyridine nitrogen. In crystal structures of (**135tfib**)(**246col**)$_3$ and (**135tfib**)(**dmap**)$_3$ two symetrically indenpendent donor molecules have been found and both such molecules form 1:3 halogen-bonded complexes. In majority of crystal structures two halogen bonds per donor molecule are related by symetry (and consequently they are of the same geometries) except those found in (**135tfib**)(**3pic**)$_3$ and one of the indenpendent molecular complexes in (**135tfib**)(**246col**)$_3$ which do have three halogen bonds of the different lengths (Table S4). It is good to notice that cocrystals of 1:3

stoichiometry have generally formed by **135tfib** and strong bases (pK_a > 5.7) with the exception of **24lut** (pK_a = 6.5) forming 1:2 cocrystal.

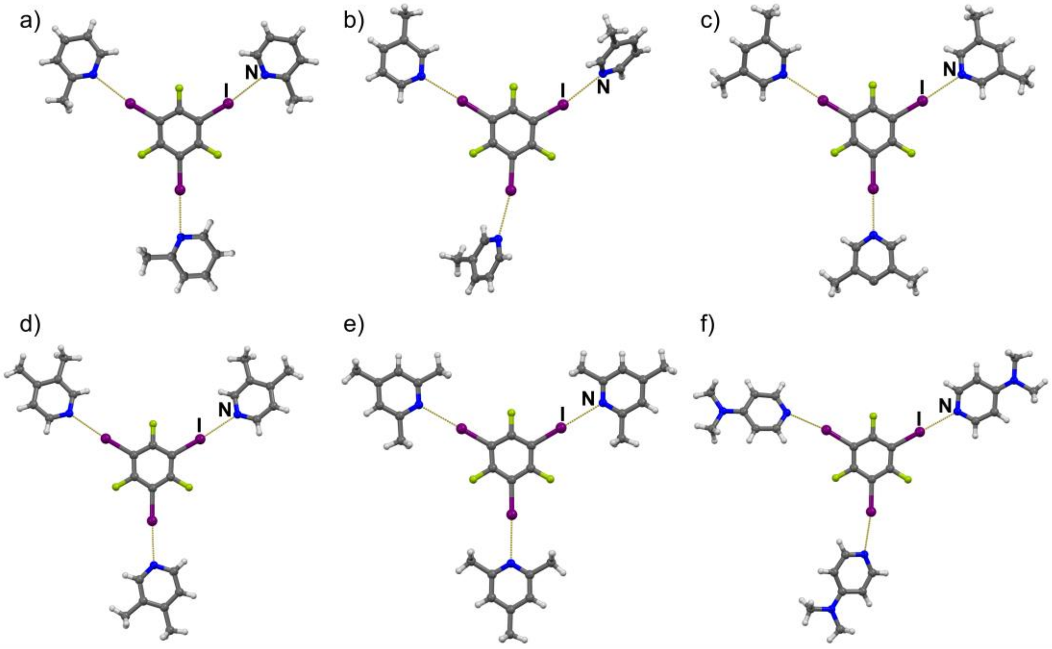

Figure S23. 1:3 halogen bonded complexes in cocrystals a) (**135titfb**)(**2pic**)$_3$ b) (**135titfb**)(**3pic**)$_3$, c) (**135titfb**)(**35lut**)$_3$, d) (**135titfb**)(**34lut**)$_3$ and e) (**135titfb**)(**246col**)$_3$ and f) (**135titfb**)(**dmap**)$_3$.

Table S4. Geometrical parameters of halogen bonds in **135tfib** cocrystals.

compound	halogen bond	*d* / Å	*r.s.* / %	ϑ(C-I···N)/°
**(135titfb)(dmap)**$_3$	I1···N1	2.896(6)	18.0	170.4(2)
	I2···N2	2.777(1)	21.3	178.1(7)
	I3···N3	2.871(7)	18.7	169.4(2)
	I4···N4	2.790(2)	21.0	176.3(7)
**(135titfb)(35lut)**$_3$	I1···N1	2.790(1)	21.0	180.0(2)
	I2···N2	2.863(8)	19.0	175.9(3)
**(135titfb)(34lut)**$_3$	I1···N1	2.836(1)	19.7	176.4(2)
	I2···N2	2.879(5)	18.4	175.4(1)
**(135titfb)(3pic)**$_3$	I1···N1	2.883(6)	18.3	177.9(2)
	I2···N2	3.044(7)	13.8	168.7(2)
	I3···N3	3.008(7)	14.8	166.5(2)
**(135titfb)(246kol)**$_3$	I1···N1	2.967(6)	15.6	178.3(2)
	I2···N2	2.985(3)	15.3	180.0(2)
	I3···N3	2.973(6)	15.8	178.0(2)
	I4···N4	2.971(6)	15.3	177.5(2)
	I5···N5	2.986(7)	15.4	176.1(2)
**(135titfb)(ikin)**$_2$	I1···N1	2.801(4)	20.7	178.0(1)
	I2···N2	2.823(5)	20.0	178.4(2)
**(135titfb)(24lut)**$_2$	I1···N1	2.814(7)	20.3	176.2(2)
	I2···N2	2.829(8)	19.9	173.0(2)
**(135titfb)(4acpy)**$_2$	I1···N1	2.863(4)	18.9	167.9(2)
**(135titfb)(acr)**	I1···N1	3.005(5)	14.9	173.7(2)
**(135titfb)(4cnpy)**	I2···N2	2.866(6)	18.8	174.8(2)
**(135titfb)(kin)**	I1···N1	2.826(5)	19.9	177.9(2)
**(135titfb)(3acpy)**	I1···N1	2.819(4)	20.1	175.7(2)

Masses and volumes of the reactants used in cocrystal synthesis

Table S5. Experimental conditions for mechanochemical synthesis of 1,4-diiodotetrafluorobenzene cocrystals.

acceptor	*m*(acceptor) / mg	*V*(acceptor) / μL	*ρ*(acceptor) / g cm^{-3}	*m*(**14tfib**) / mg	stoichiometric ratio D:A
**2pic**	-	33.3	0.943	68.5	1:2
**3pic**	-	33.3	0.957	68.5	1:2
**4pic**	-	33.2	0.957	68.5	1:2
**24lut**	-	37.9	0.927	67.2	1:2
**26lut**	-	38.6	0.920	67.2	1:2
**34lut**	-	37.2	0.954	67.2	1:2
**35lut**	-	37.0	0.939	65.2	1:2
**246col**	-	40.1	0.917	62.9	1:2
**3acpy**	-	34.8	1.102	64.3	1:2
**4acpy**	-	35.4	1.095	64.3	1:2
**qin**	-	36.0	1.090	60.6	1:2
**iqin**	-	35.5	1.100	60.6	1:2
**4cnpy**	34.8	-	-	67.9	1:2
**acr**	29.1	-	-	19.4	1:2

Table S6. Experimental conditions for mechanochemical synthesis of 1,3,5-trifluoro-2,4,6-triiodobenzene cocrystals.

acceptor	*m*(acceptor) / mg	*V*(acceptor) / μL	*ρ*(acceptor) / g cm^{-3}	*m*(**135tftib**) / mg	stoichiometric ratio D:A
**2pic**	-	38.3	0.943	64.6	1:3
**3pic**	-	37.7	0.957	64.6	1:3
**3pic**	-	28.4	0.957	72.8	1:2
**3pic**	-	16.4	0.957	84.3	1:1
**4pic**	-	38.1	0.957	64.6	1:3
**24lut**	-	42.1	0.927	61.3	1:3
**26lut**	-	42.9	0.920	61.3	1:3
**34lut**	-	41.4	0.954	61.3	1:3
**34lut**	-	30.0	0.954	70.0	1:2
**34lut**	-	18.5	0.954	82.3	1:1
**35lut**	-	42.5	0.939	61.3	1:3
**35lut**	-	32.2	0.939	69.8	1:2
**35lut**	-	19.0	0.939	82.2	1:1
**246col**	-	45.9	0.917	58.4	1:3
**246col**	-	35.4	0.917	67.6	1:2
**246col**	-	21.2	0.917	80.6	1:1
**3acpy**	-	38.5	1.102	58.4	1:3
**4acpy**	-	39.2	1.095	58.4	1:3
**qin**	-	41.2	1.090	56.8	1:3
**iqin**	-	40.5	1.100	56.8	1:3
**4cnpy**	38.8	-	-	62.0	1:3

Table S7. Experimental conditions for mechanochemical synthesis of 1,3-diiodotetrafluorobenzene cocrystals.

acceptor	*m*(acceptor) / mg	*V*(acceptor) / μL	*ρ*(acceptor) / g cm^{-3}	*V*(**13tfib**) / μL	stoichiometric ratio D:A
**2pic**	-	34.3	0.943	25.1	1:2
**3pic**	-	33.8	0.957	25.1	1:2
**4pic**	-	34.1	0.957	25.1	1:2
**24lut**	-	37.9	0.927	23.9	1:2
**26lut**	-	38.6	0.920	23.9	1:2
**34lut**	-	37.2	0.954	23.9	1:2
**35lut**	-	38.2	0.939	23.9	1:2
**246col**	-	41.4	0.917	22.9	1:2
**3acpy**	-	34.8	1.102	22.9	1:2
**4acpy**	-	35.4	1.095	22.9	1:2
**qin**	-	37.3	1.090	22.3	1:2
**iqin**	-	36.7	1.100	22.3	1:2
**4cnpy**	31.2	-	-	24.2	1:2
**acr**	29.1	-	-	19.4	1:2

XRPD patterns of the prepared compounds

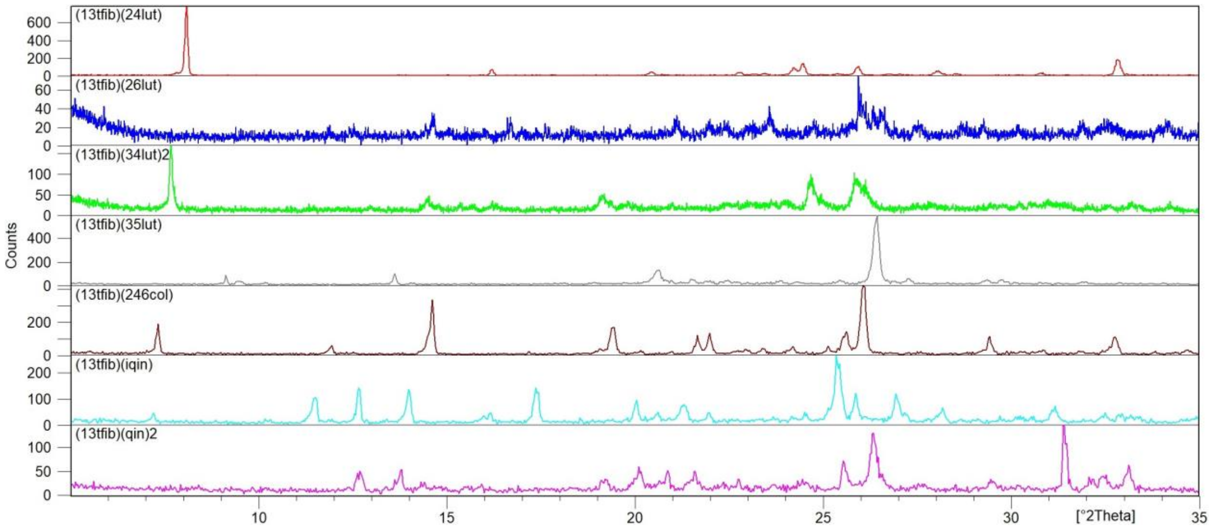

Figure S24. XRPD patterns of the **13tfib** cocrystals with the liquid acceptors. **26lut** – red, **34lut** – blue, **35lut** – green, **246col** – grey, **iqin** – brown, **qin** – light blue.

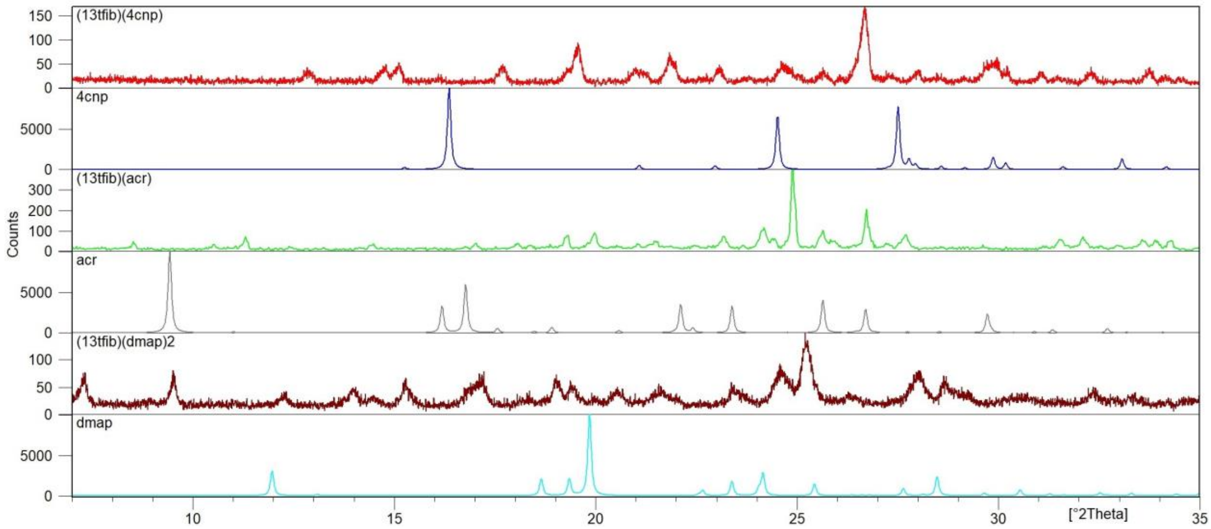

Figure S25. Comparison of the XRPD patterns of the **13tfib** cocrystals with the solid acceptors and the XRPD patterns of the pure acceptors.

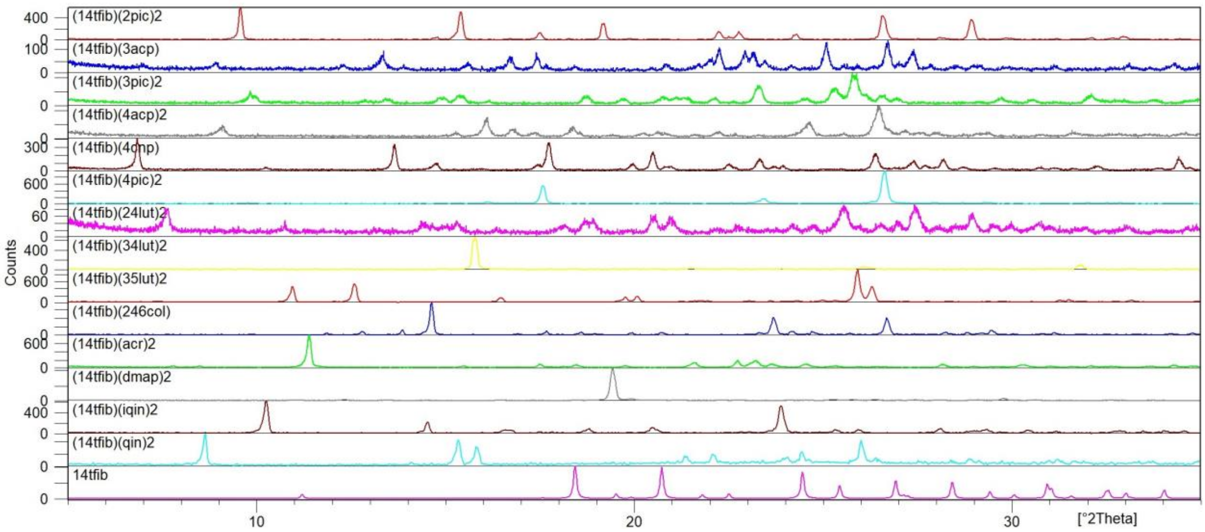

Figure S26. Comparison of the XRPD patterns of the **14tfib** cocrystals and the XRPD pattern of the **14tfib**.

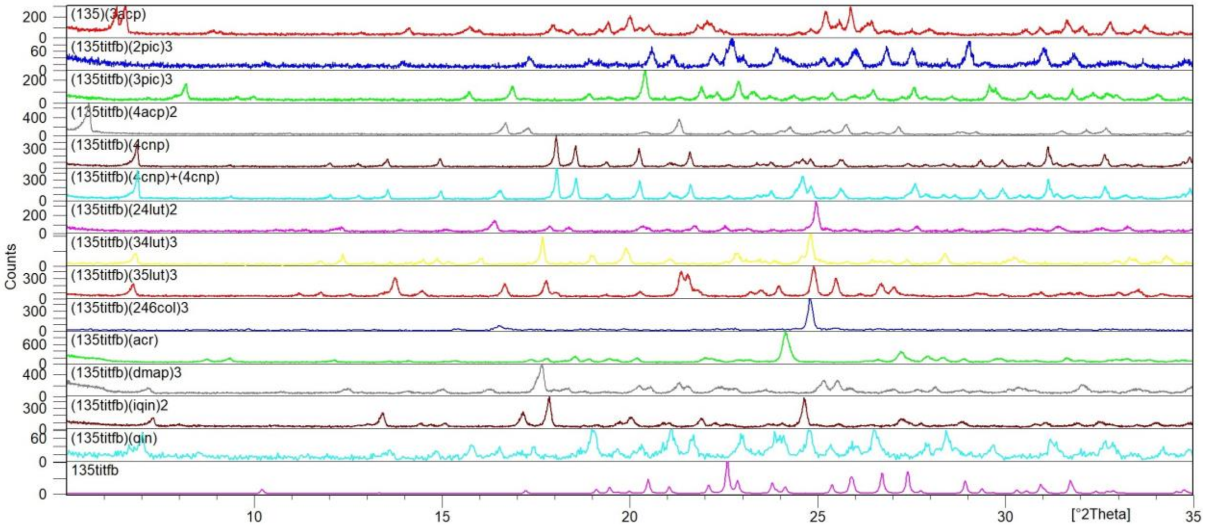

Figure S27. Comparison of the XRPD patterns of the **135titfb** cocrystals and the XRPD pattern of the **135titfb**.

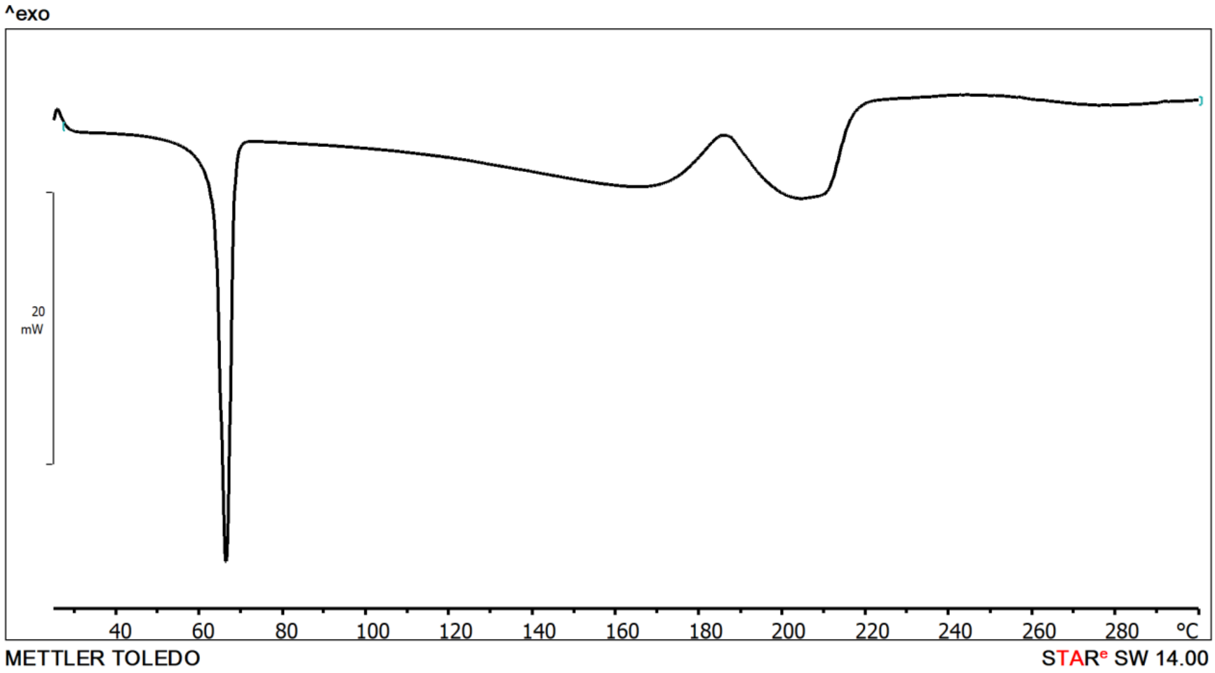

Figure D28. DSC thermogram of the (**13tfib**)(**24lut**).

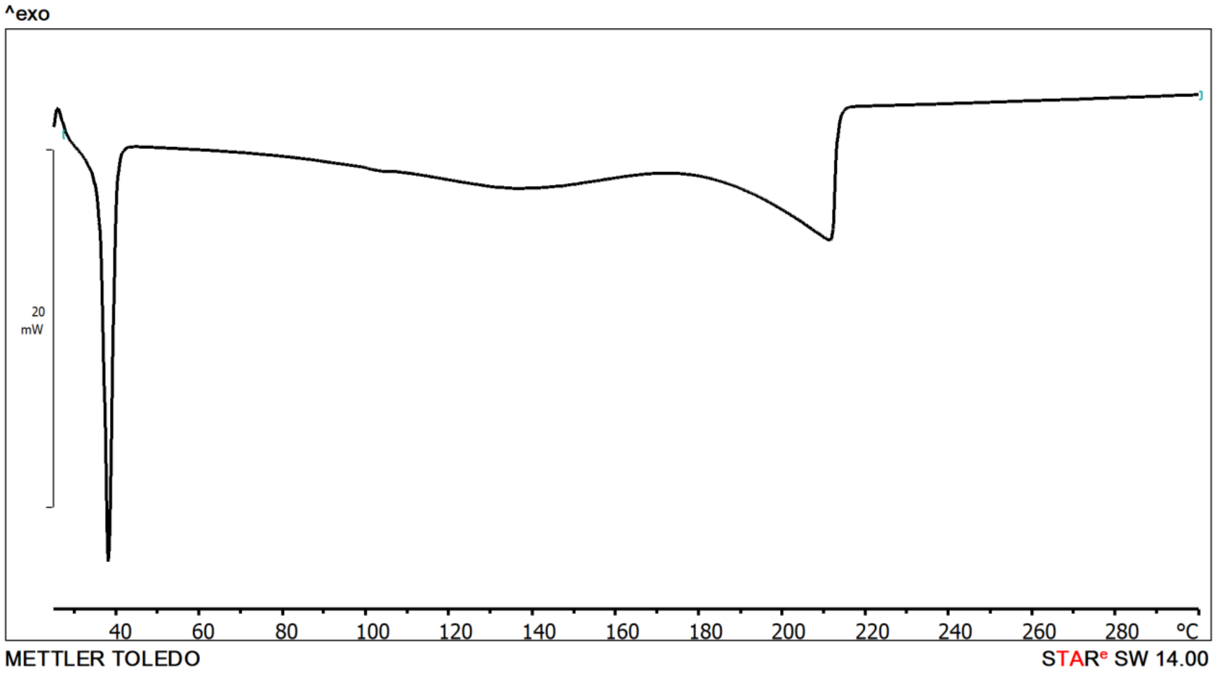

Figure D29. DSC thermogram of the (**13tfib**)(**26lut**).

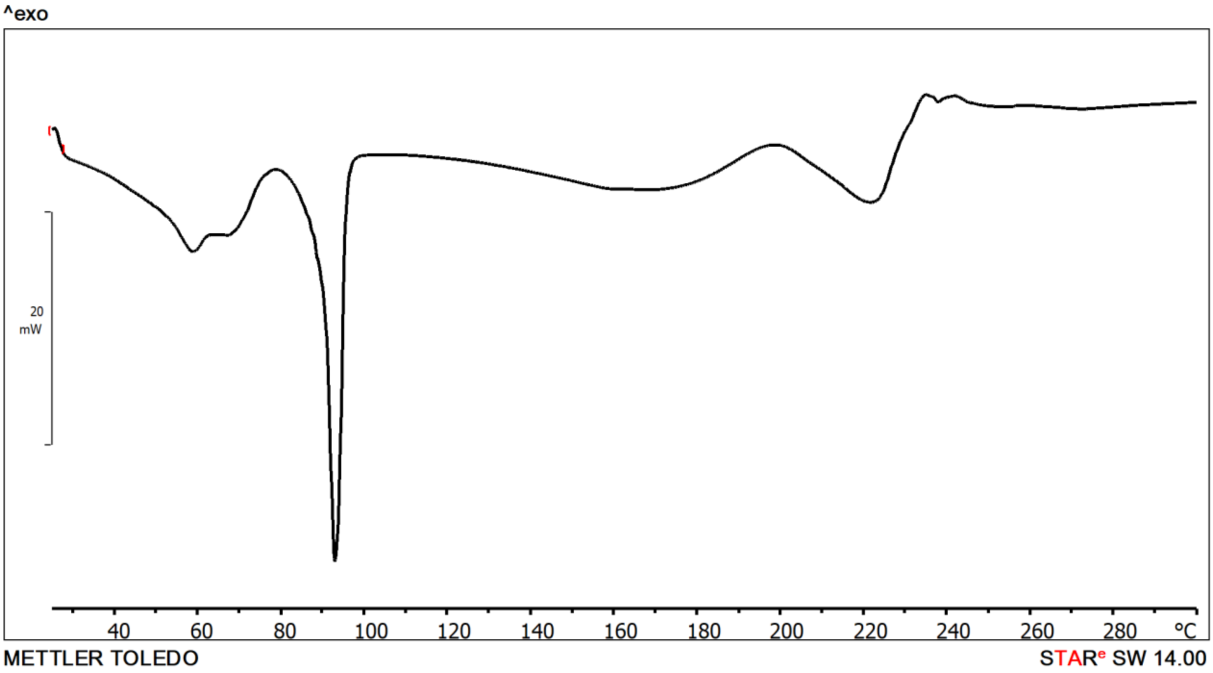

Figure D30. DSC thermogram of the (**13tfib**)(**35lut**).

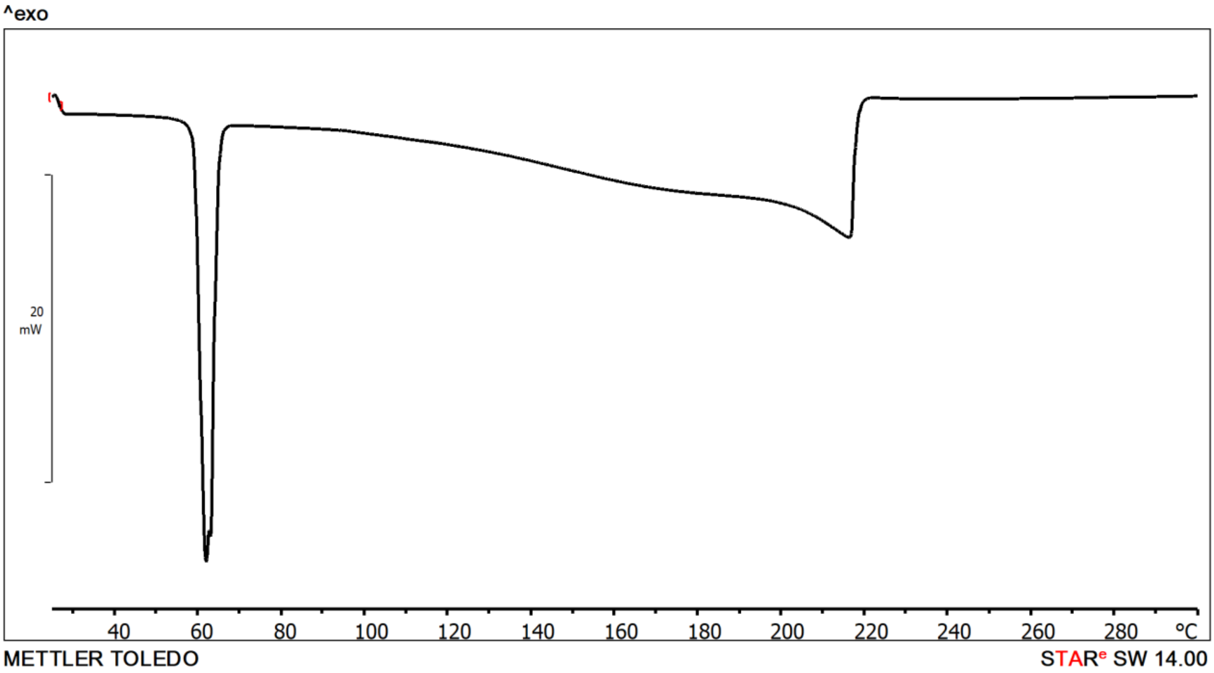

Figure D31. DSC thermogram of the (**13tfib**)(**246col**).

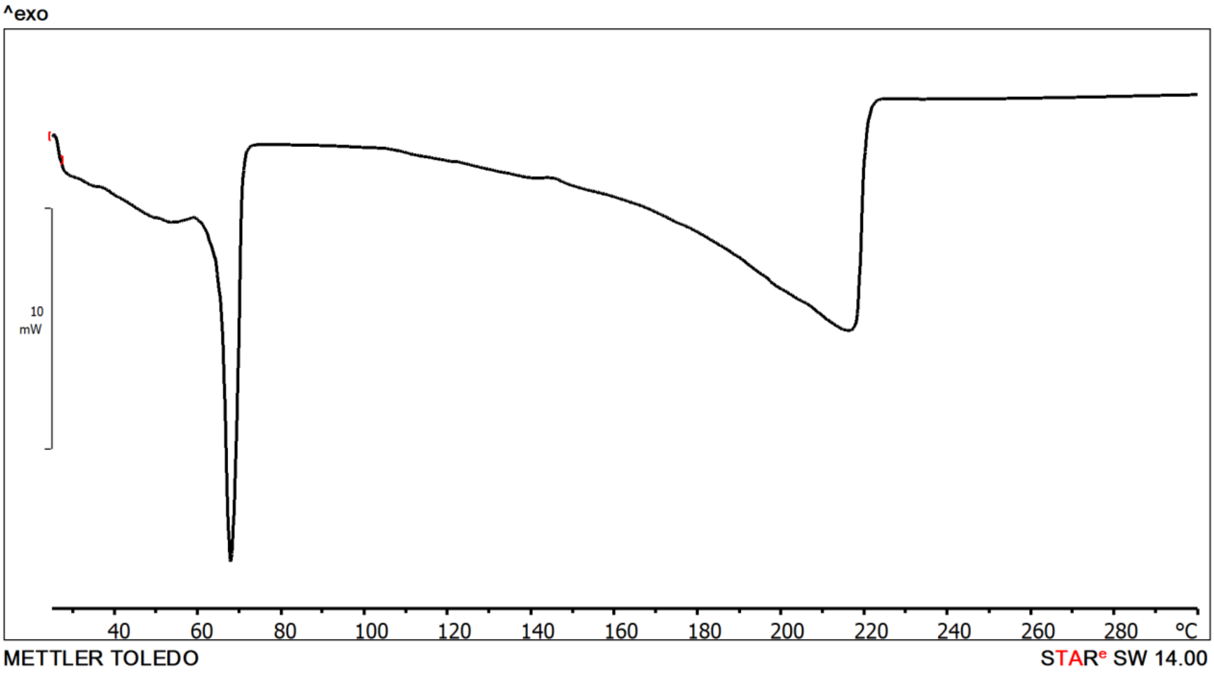

Figure D32. DSC thermogram of the (**13tfib**)(**qin**).

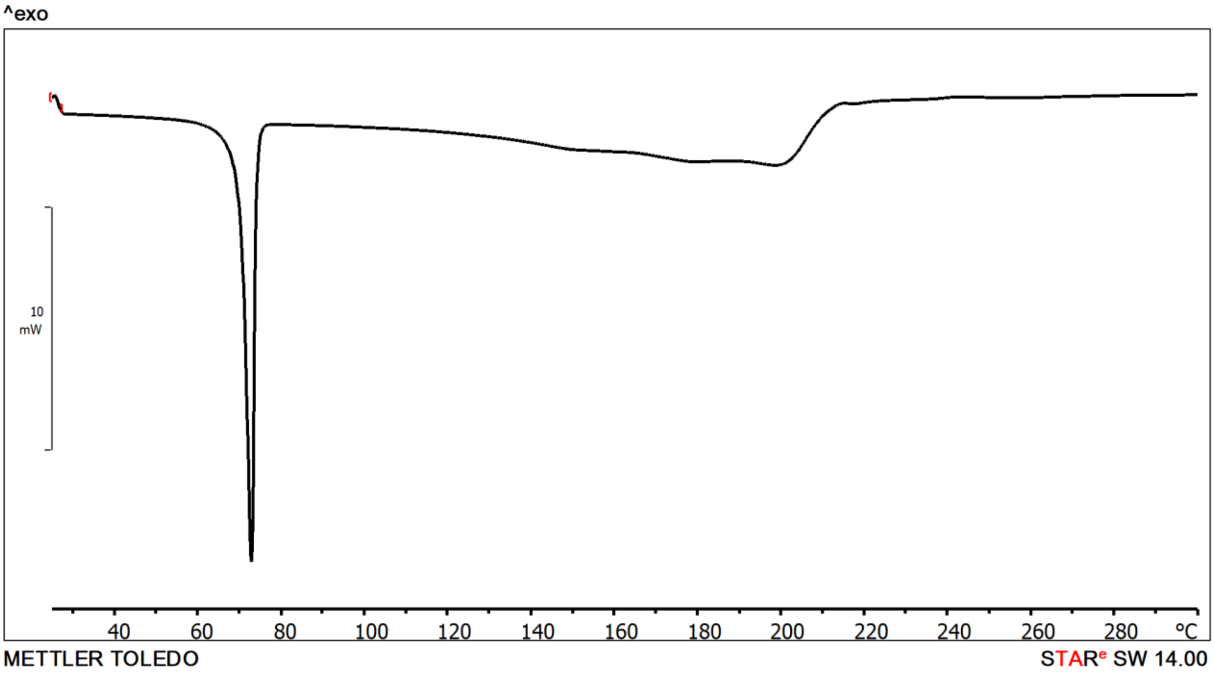

Figure D33. DSC thermogram of the (**13tfib**)(**iqin**).

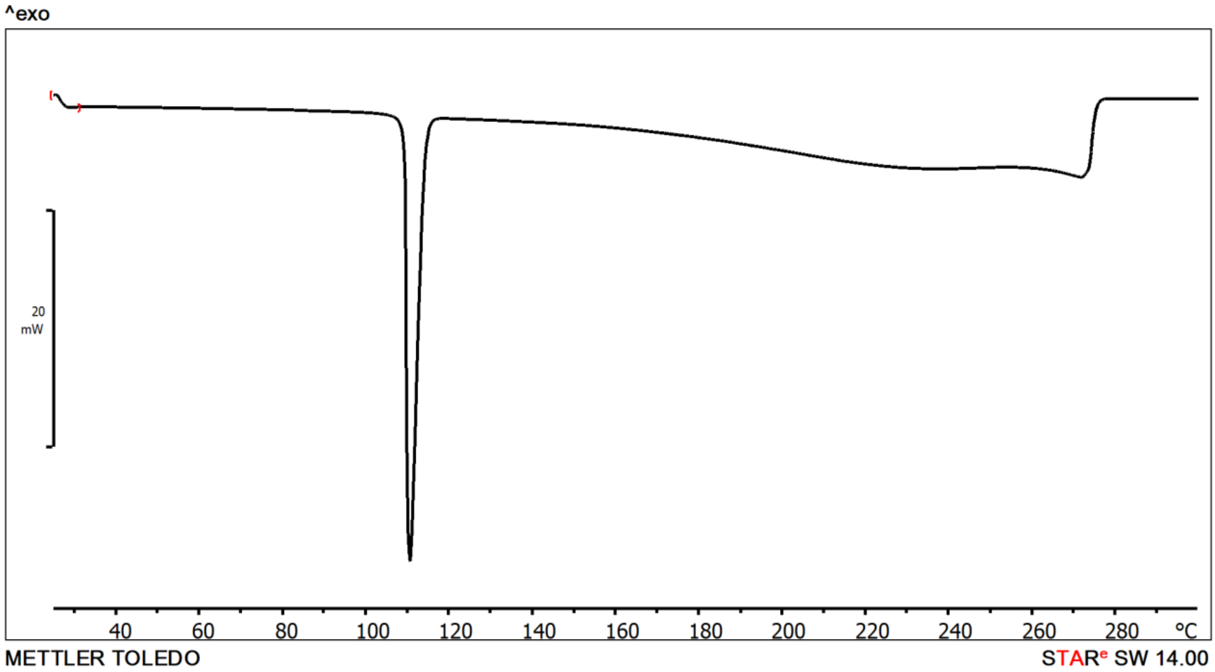

Figure D34. DSC thermogram of the (**13tfib**)(**acr**).

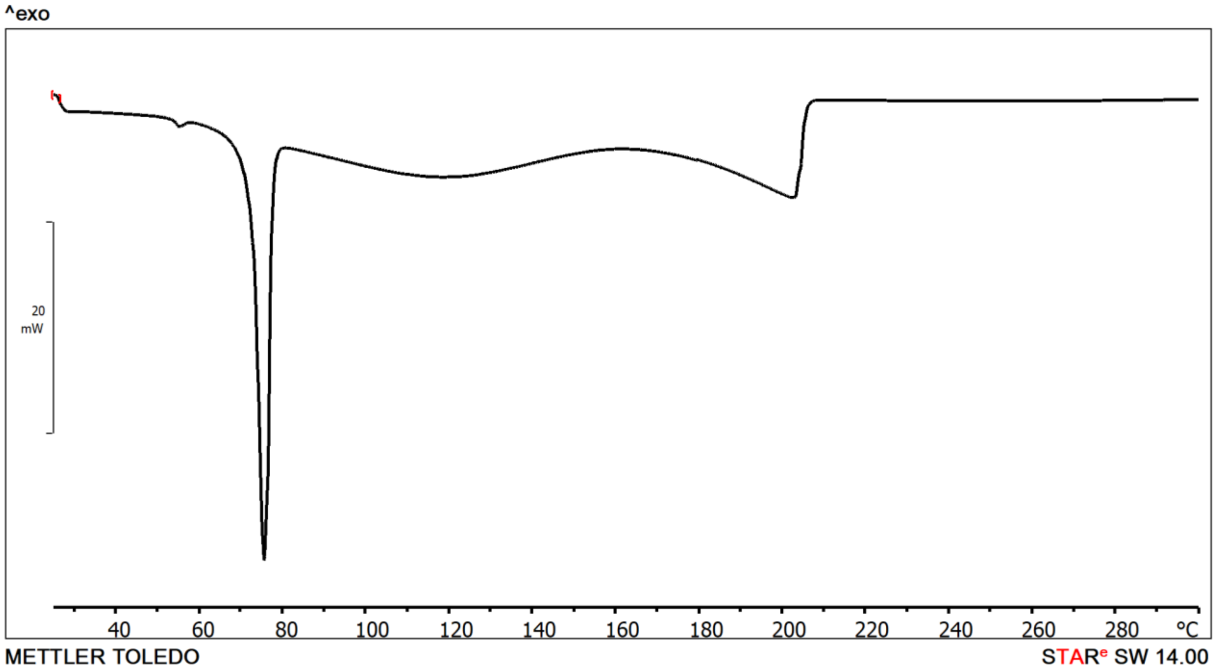

Figure D35. DSC thermogram of the (**14tfib**)(**2pic**)$_2$.

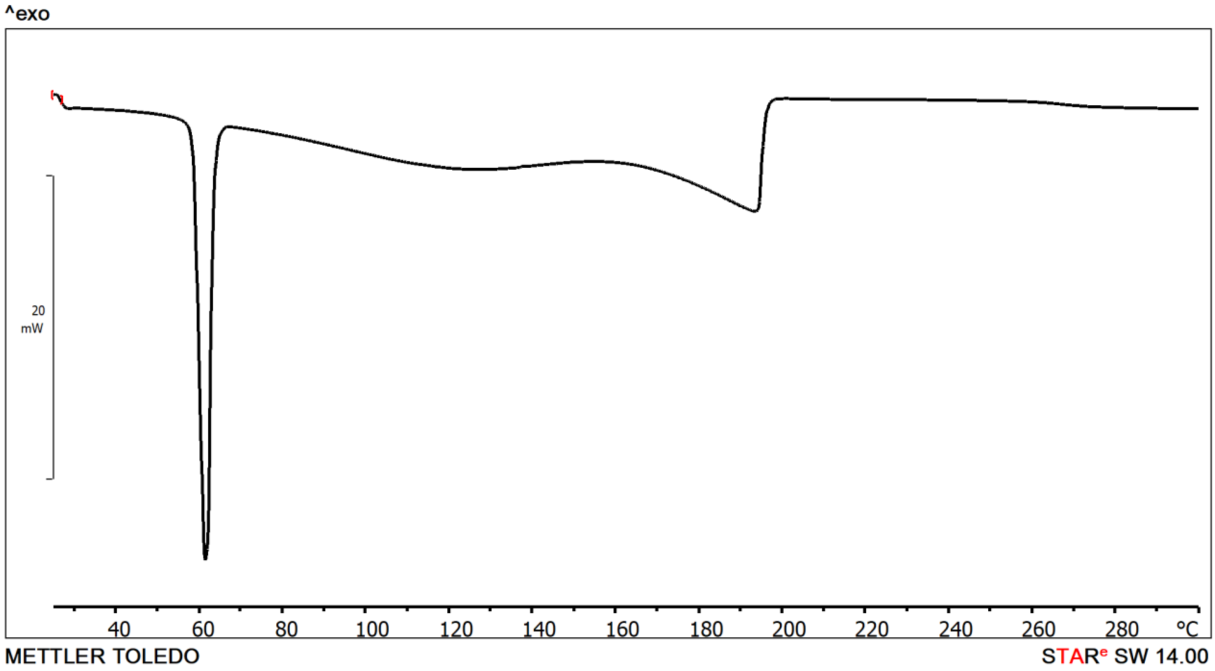

Figure D36. DSC thermogram of the (**14tfib**)(**3pic**)$_2$.

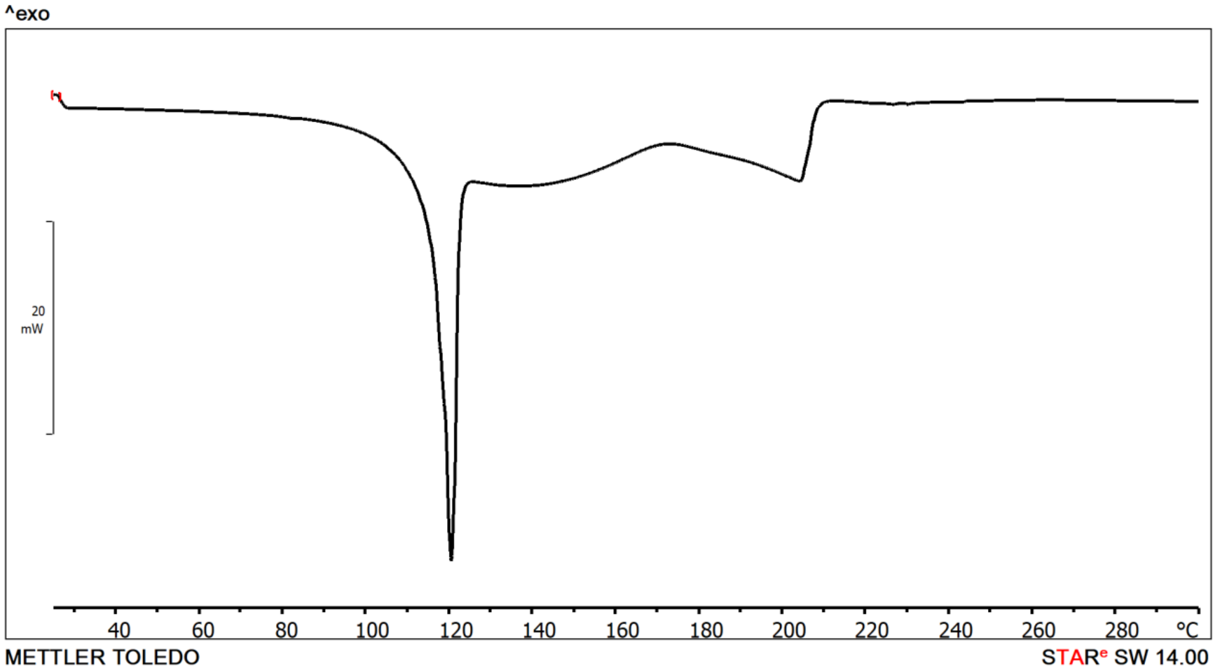

Figure D37. DSC thermogram of the (**14tfib**)(**4pic**)$_2$.

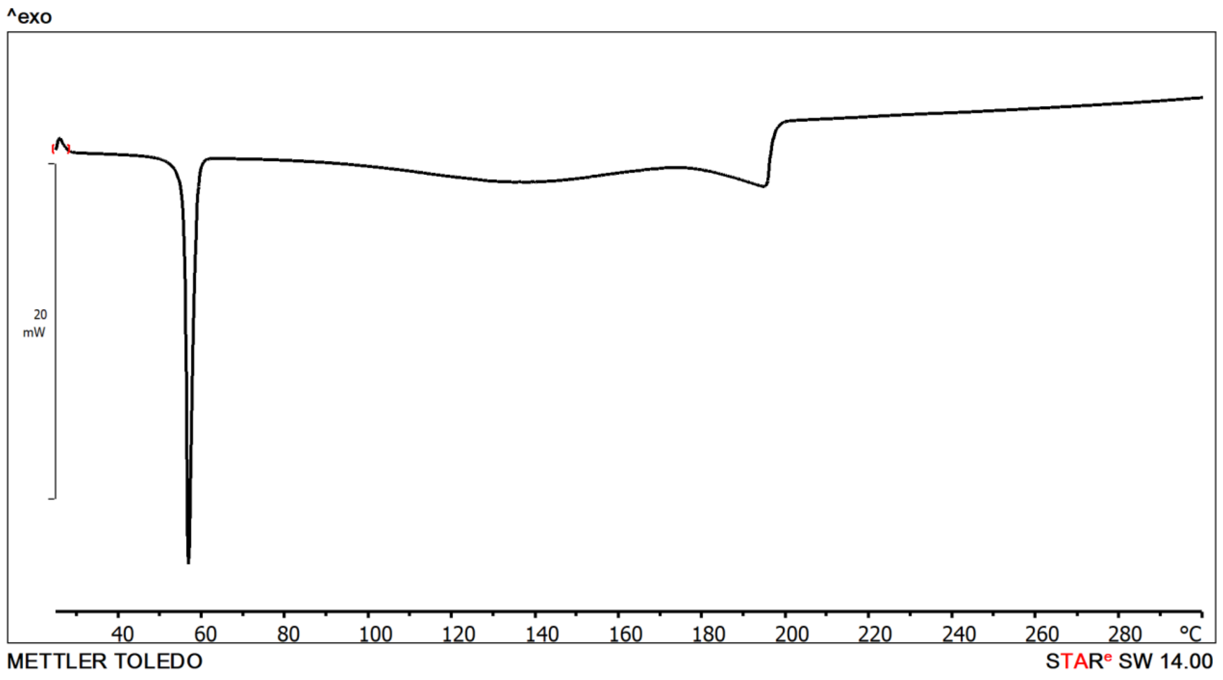

Figure D38. DSC thermogram of the (**14tfib**)(**24lut**)$_2$.

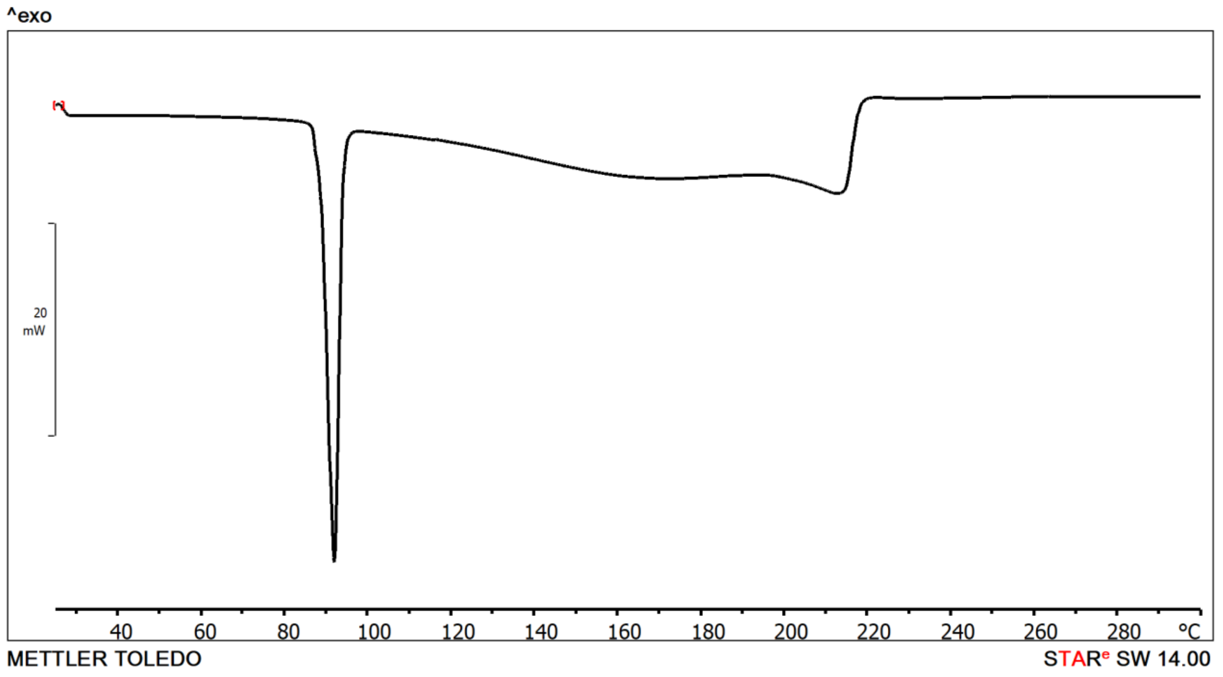

Figure D39. DSC thermogram of the (**14tfib**)(**35lut**)$_2$.

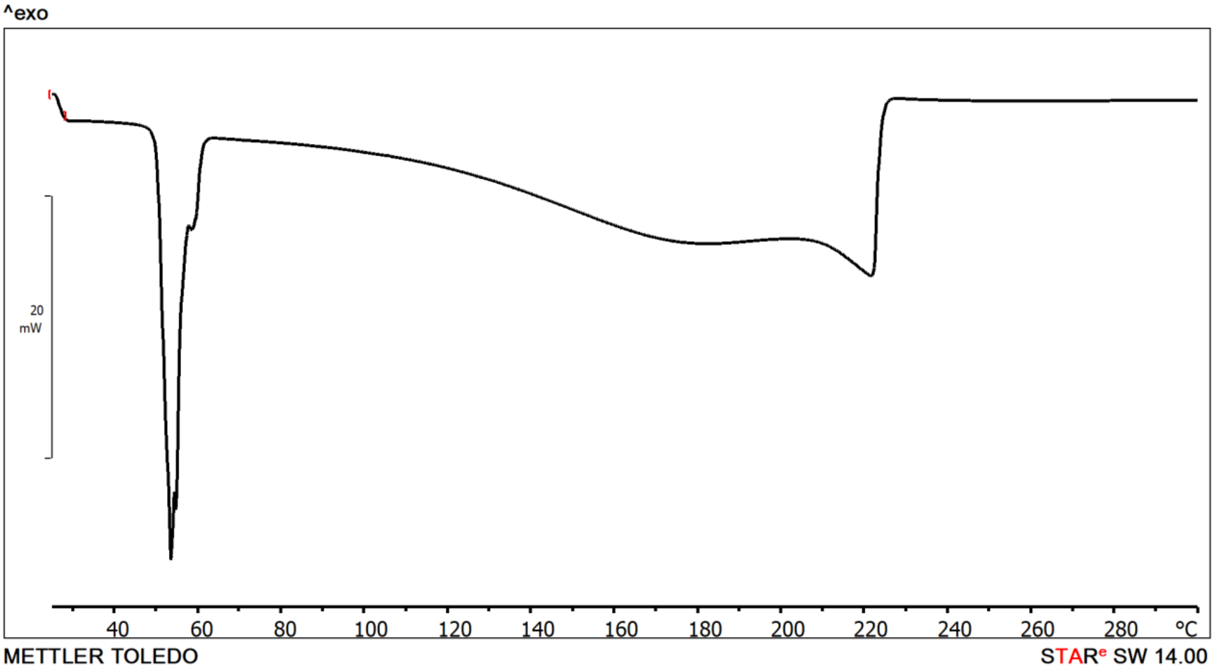

Figure D40. DSC thermogram of the (**14tfib**)(**246col**).

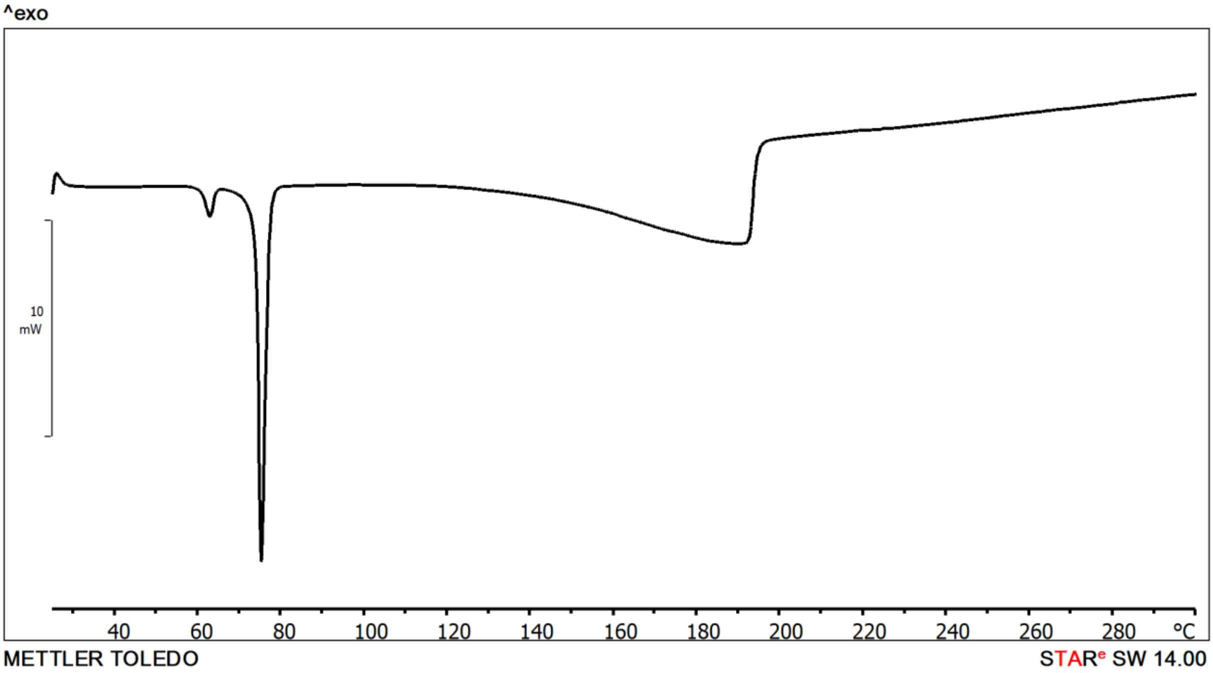

Figure D41. DSC thermogram of the (**14tfib**)(**3acpy**)$_2$.

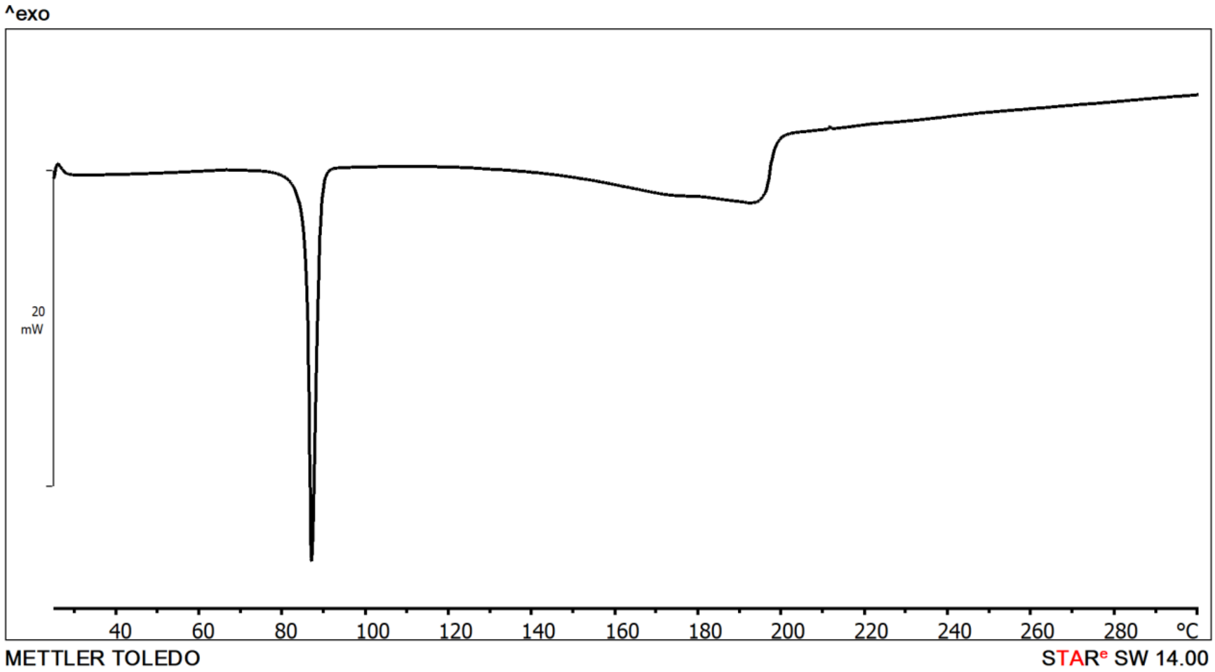

Figure D42. DSC thermogram of the (**14tfib**)(**4acpy**)$_2$.

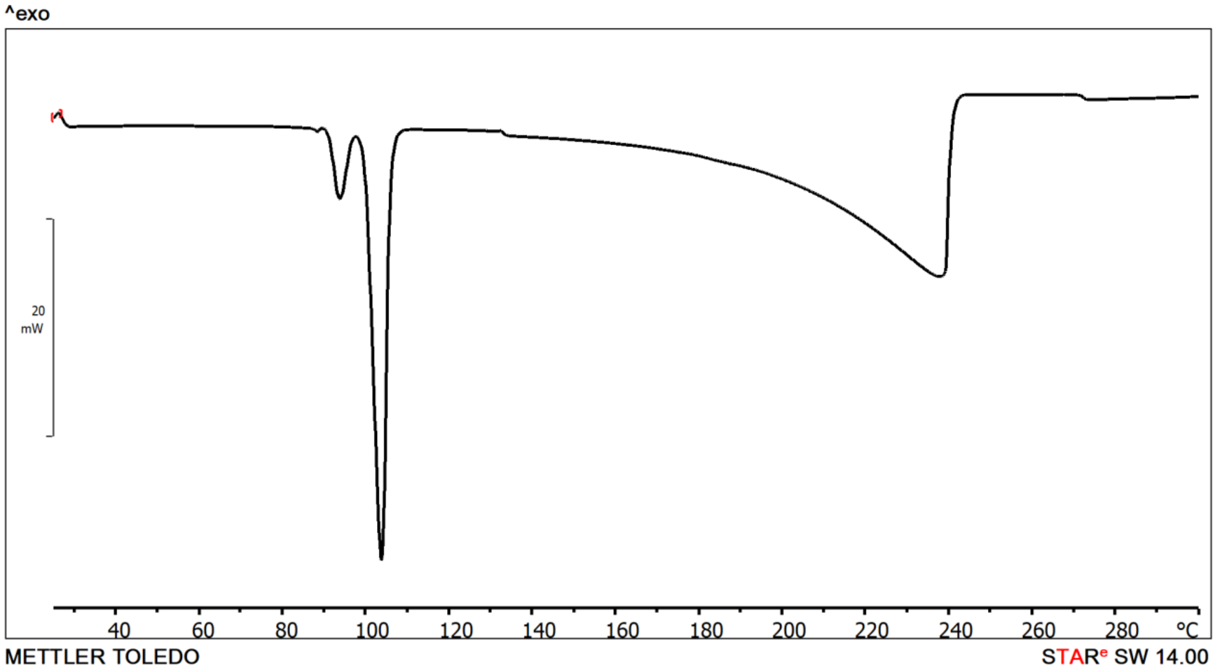

Figure D43. DSC thermogram of the (**14tfib**)(**qin**)$_2$.

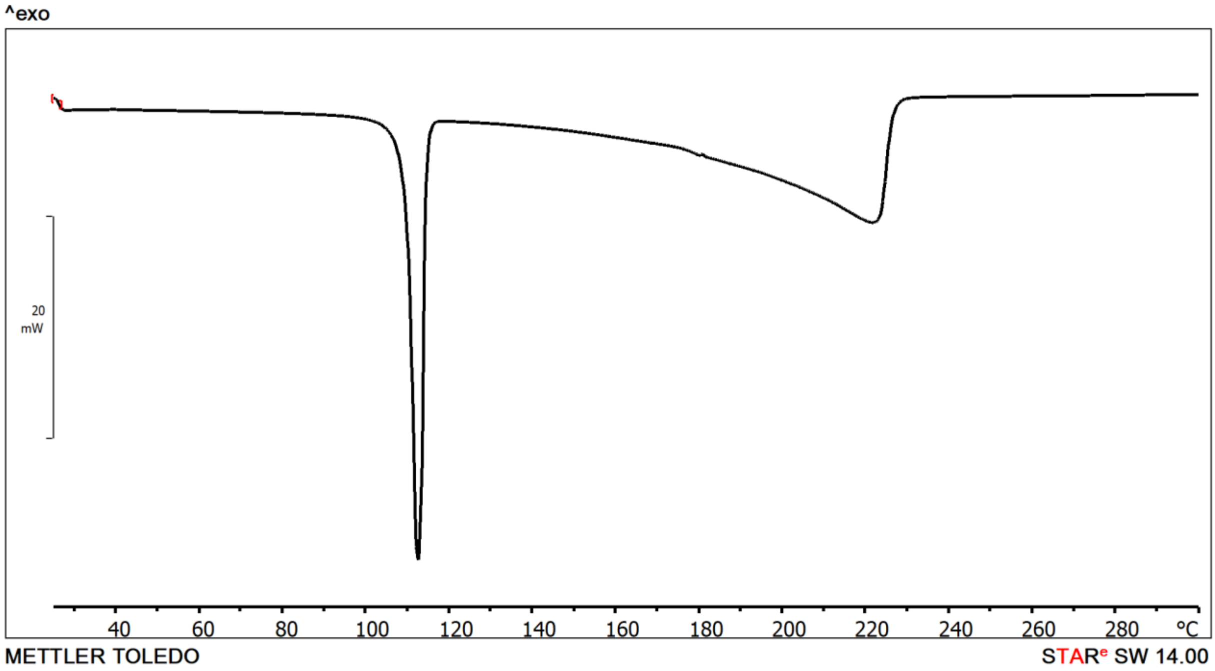

Figure D44. DSC thermogram of the (**14tfib**)(**ikin**)$_2$.

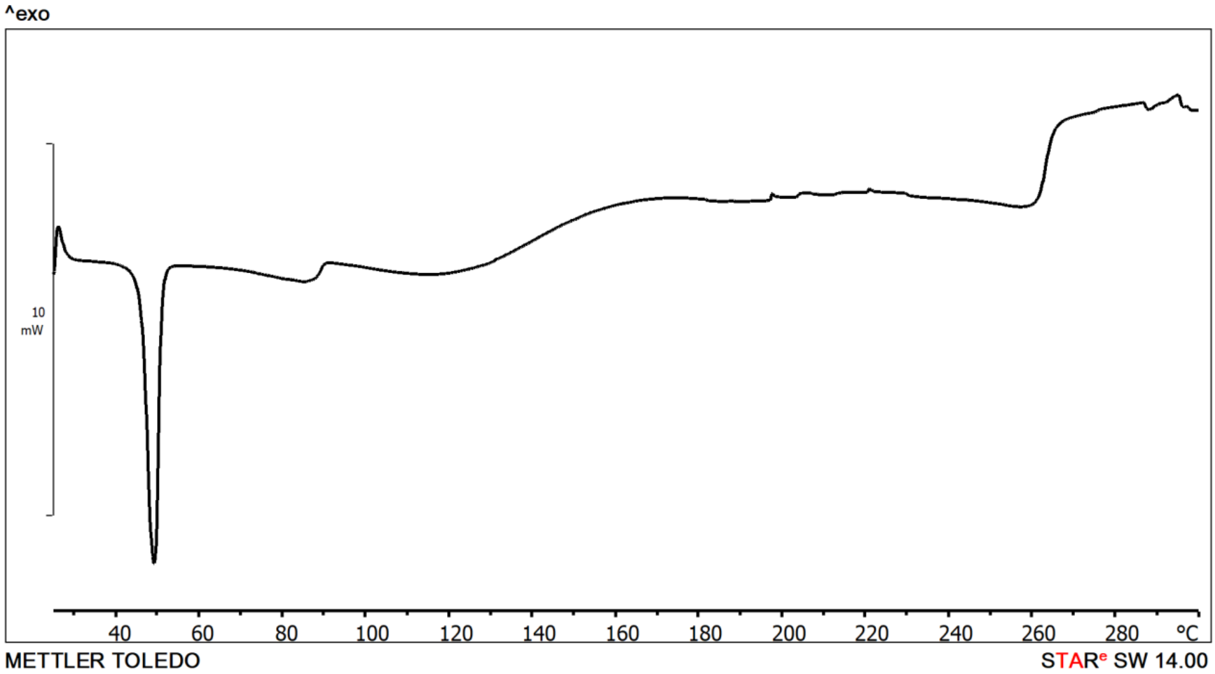

Figure D45. DSC thermogram of the (**135titfb**)(**3pic**)$_2$.

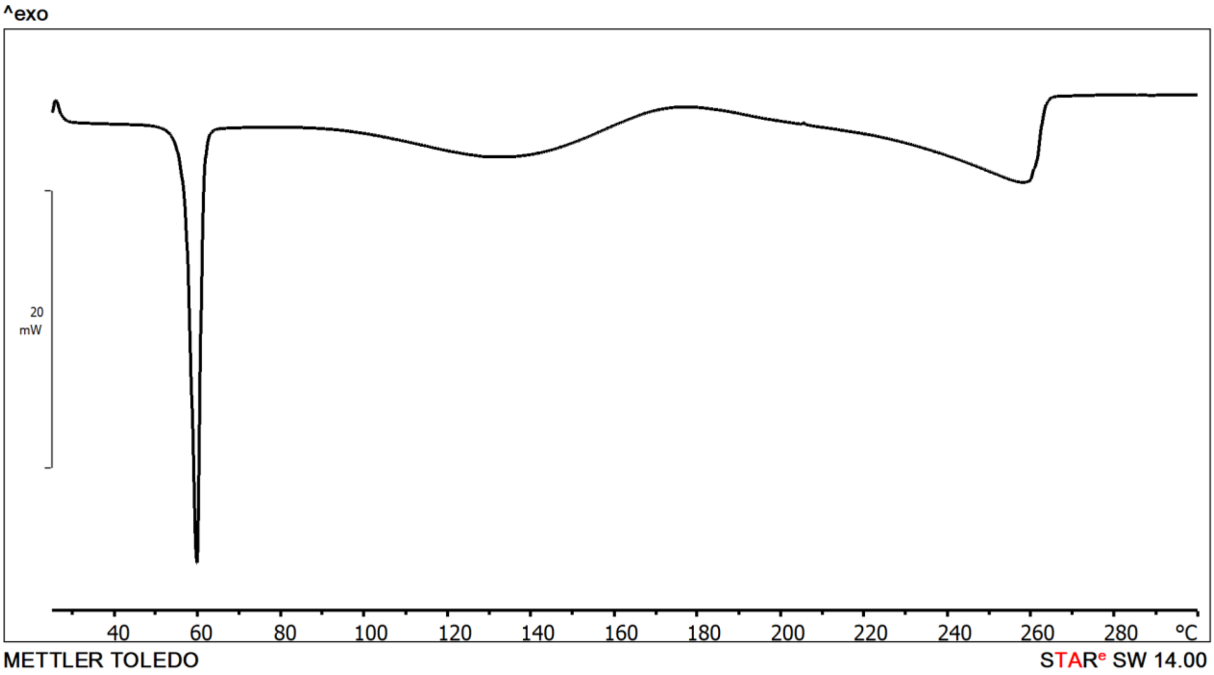

Figure D46. DSC thermogram of the (**135titfb**)(**24lut**)$_2$.

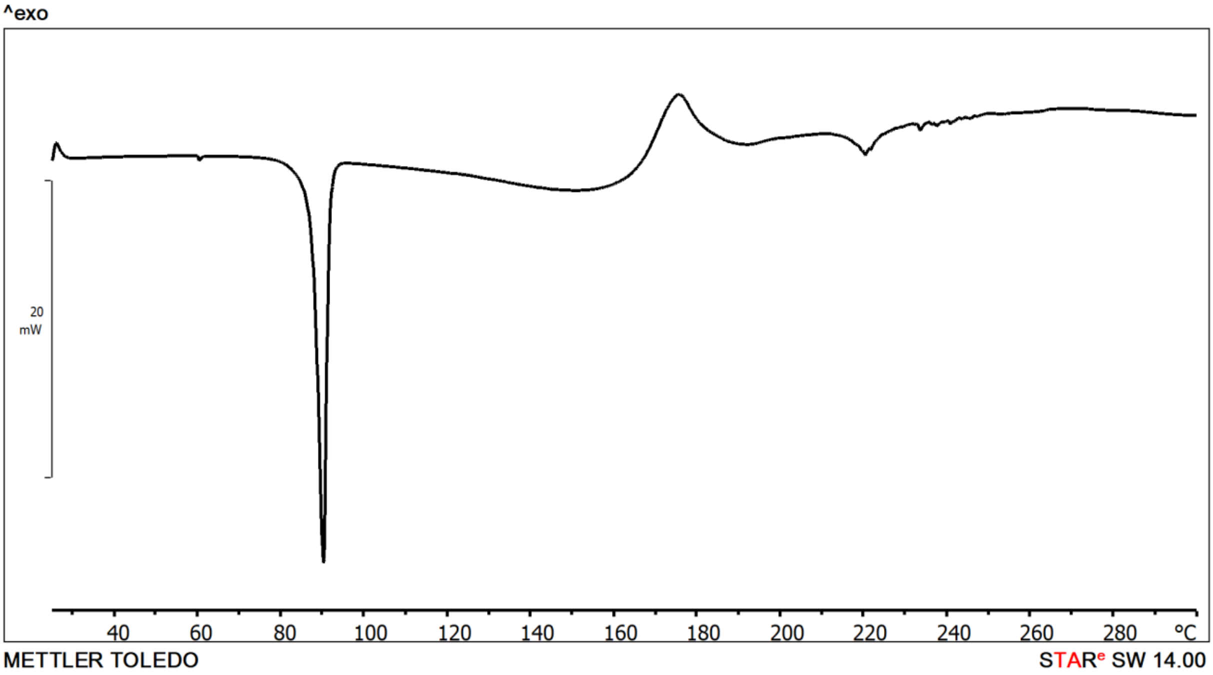

Figure D47. DSC thermogram of the (**135titfb**)(**34lut**)$_3$.

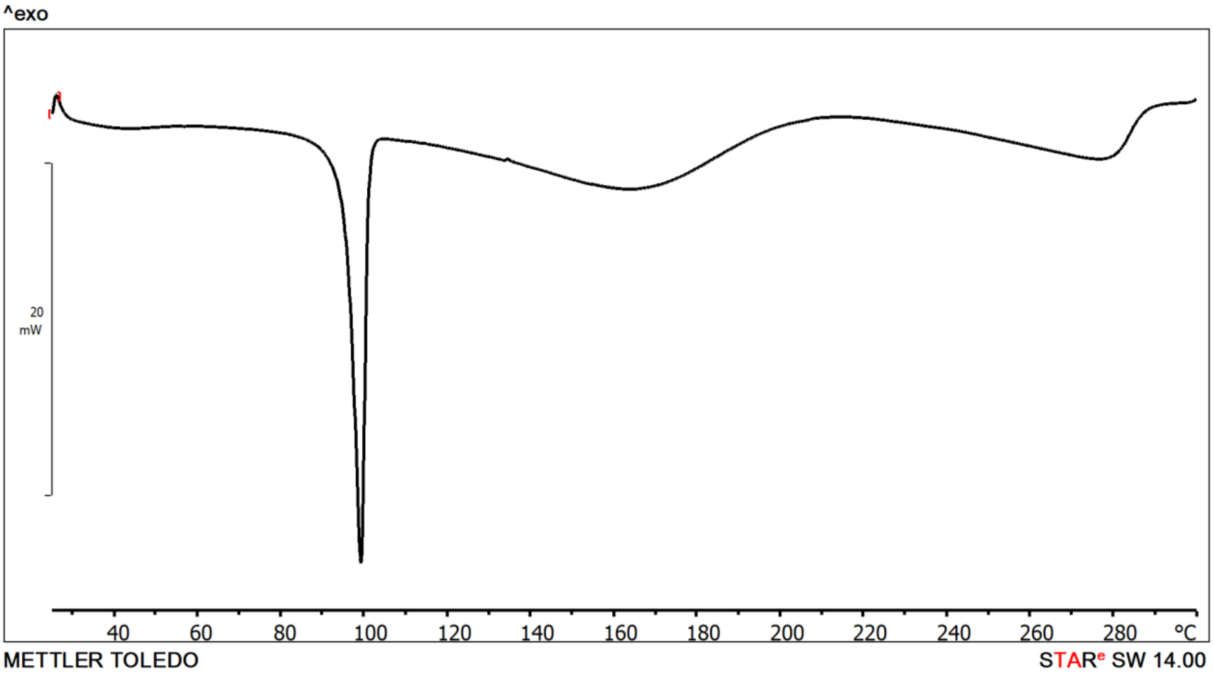

Figure D48. DSC thermogram of the (**135titfb**)(**35lut**)$_3$.

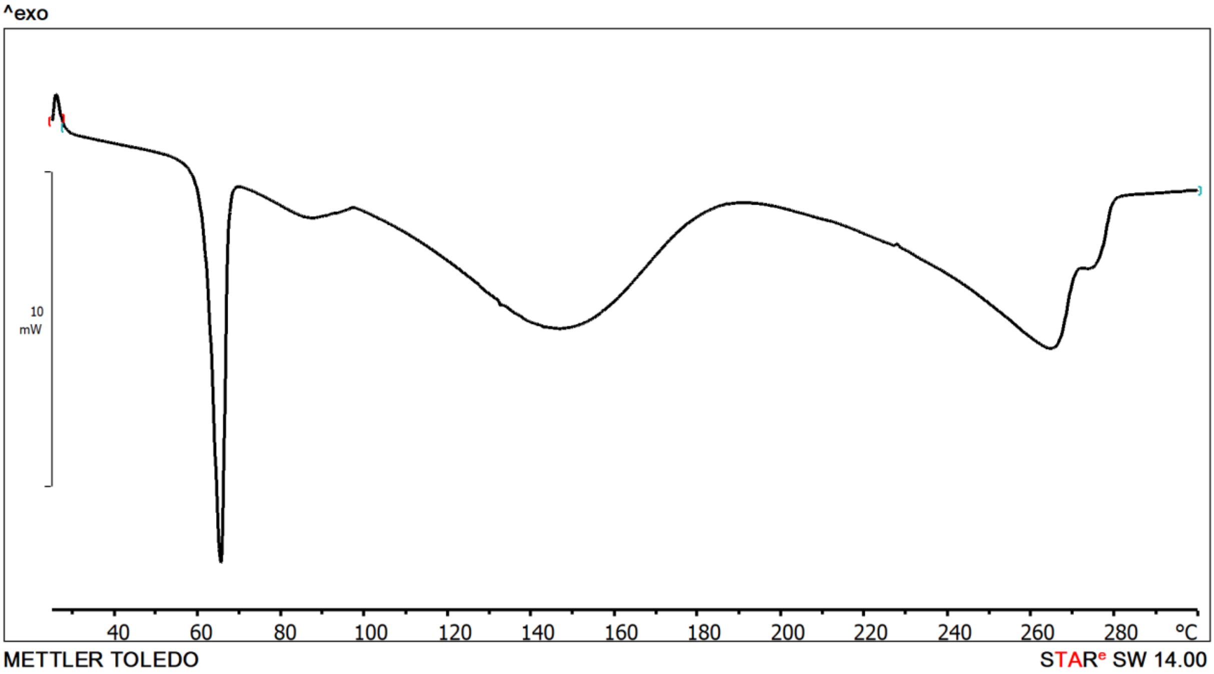

Figure D49. DSC thermogram of the (**135titfb**)(**246col**)$_3$.

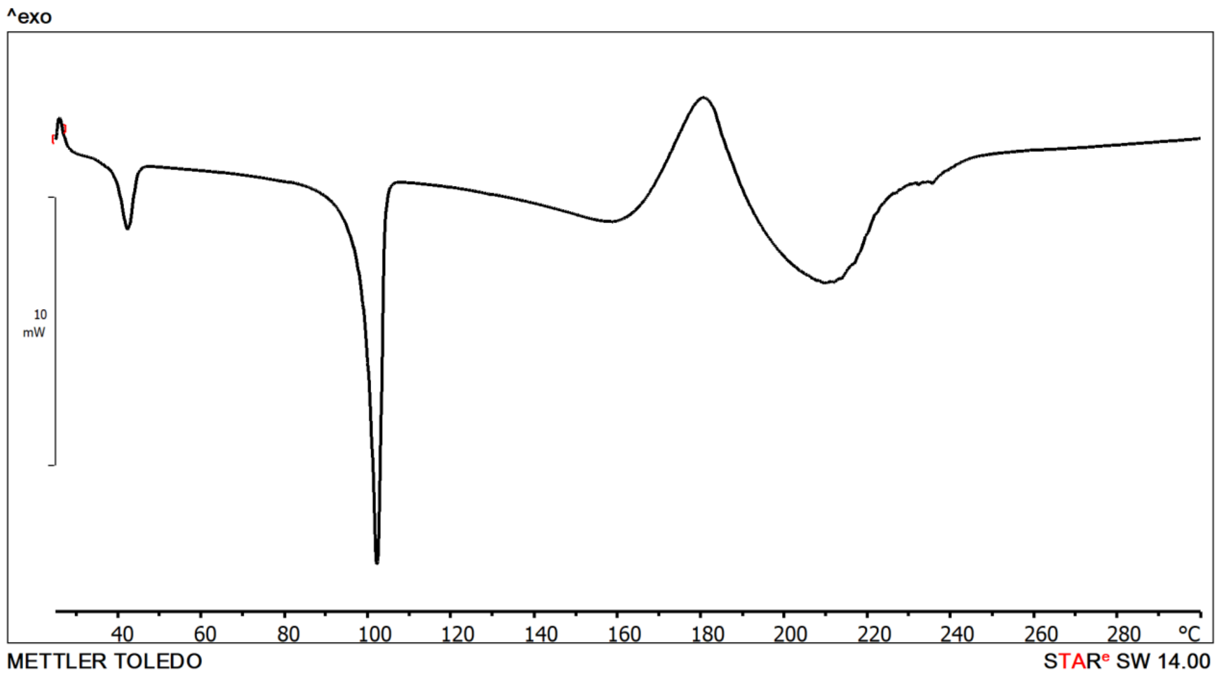

Figure D50. DSC thermogram of the (**135titfb**)(**3acpy**).

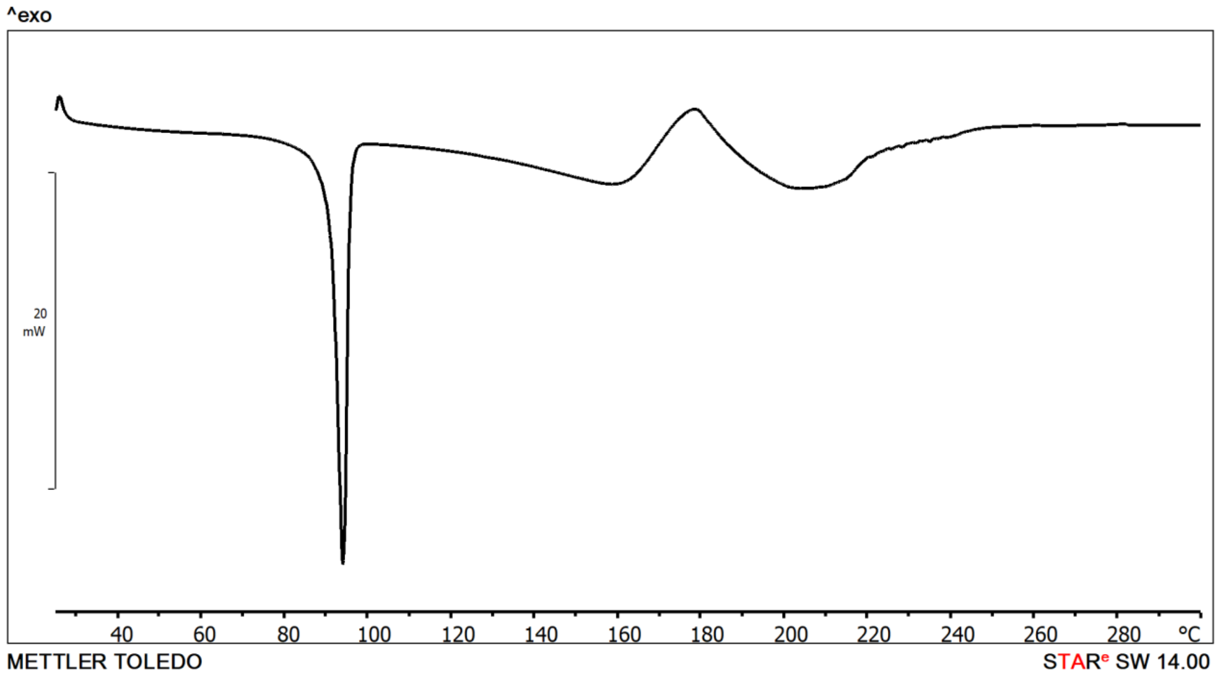

Figure D51. DSC thermogram of the (**135titfb**)(**4acpy**)$_2$.

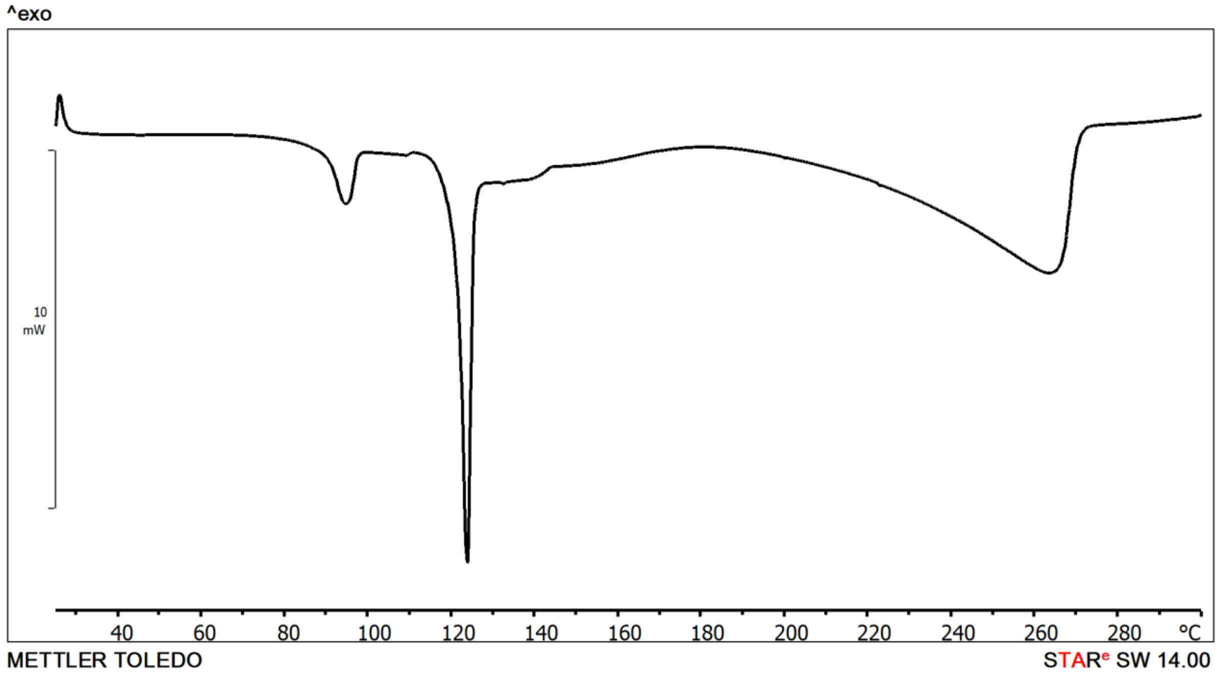

Figure D52. DSC thermogram of the (**135titfb**)(**4cnpy**).

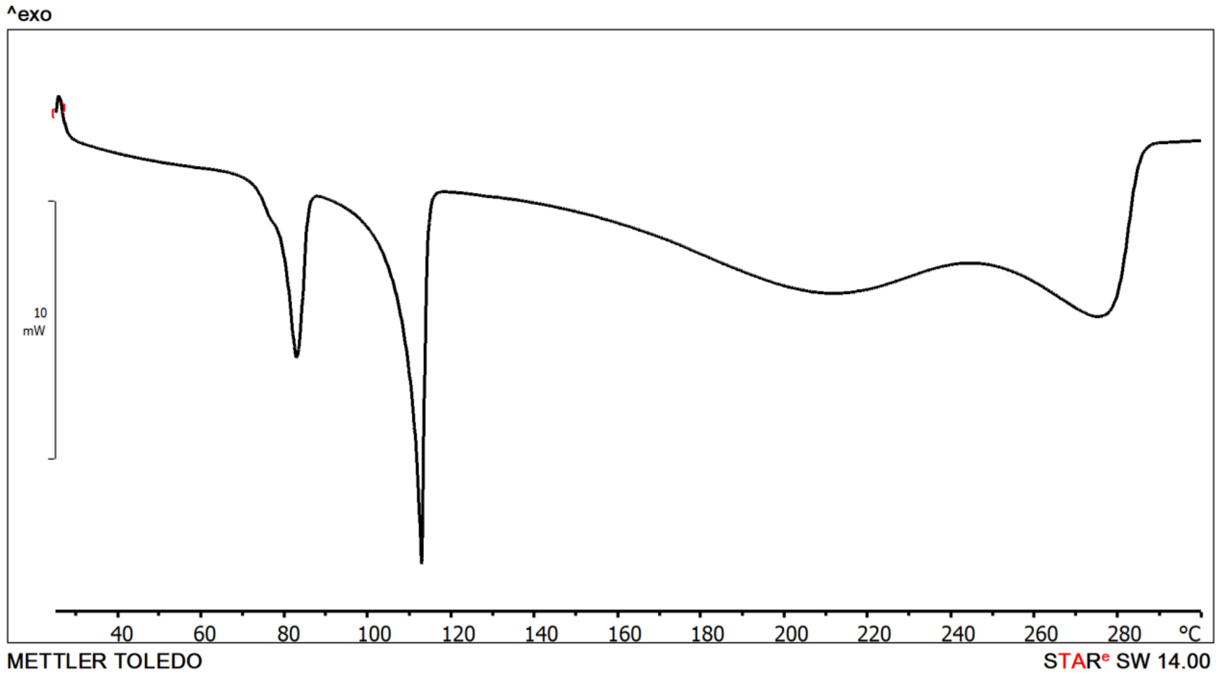

Figure D53. DSC thermogram of the (**135titfb**)(**qin**).

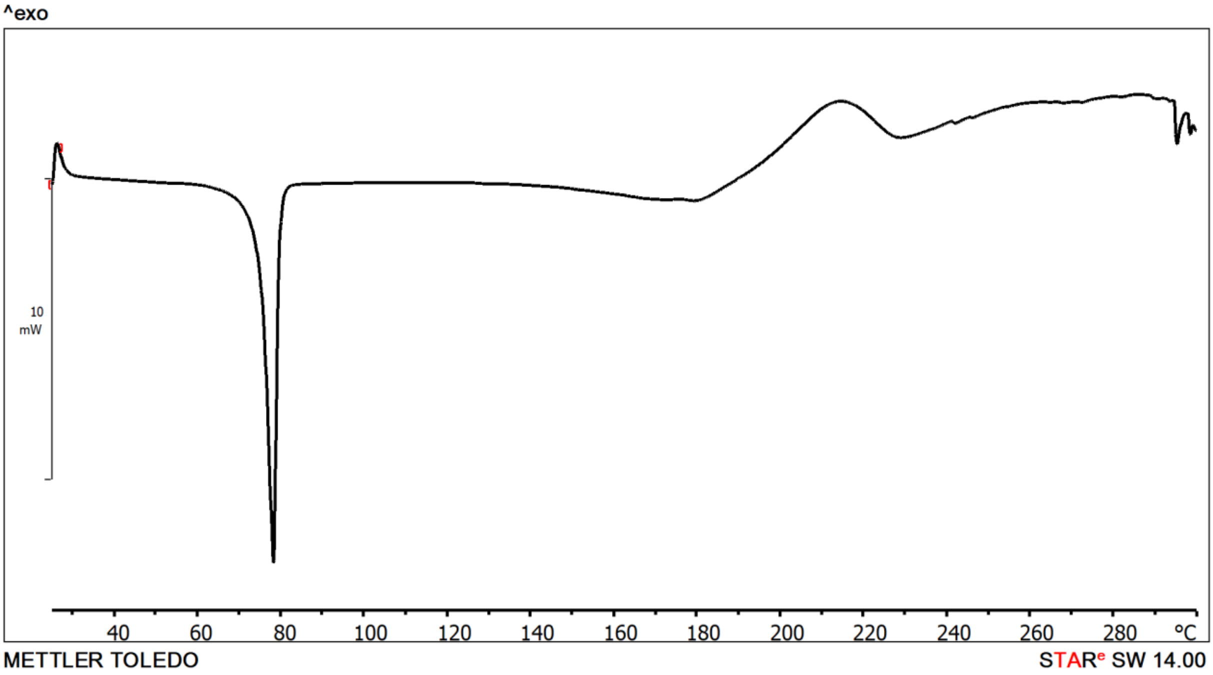

Figure D54. DSC thermogram of the (**135titfb**)(**iqin**)$_2$.
